# Dietary intake and circulating levels of vitamins, antioxidants, and fatty acids in relation to coronary heart disease risk: a systematic review and dose–response meta-analysis

**DOI:** 10.3389/fnut.2026.1920716

**Published:** 2026-10-01

**Authors:** Peng Tian, Chang Su, Yang Yang, Zhao Su, Mengrou Gao, Xiangru Tan, Qiuyan Zhang

**Affiliations:** Hunan Provincial Key Laboratory of Traditional Chinese Medicine Diagnostics, School of Traditional Chinese Medicine, Hunan University of Chinese Medicine, Changsha, China

**Keywords:** antioxidants, coronary heart disease, dose–response meta-analysis, fatty acids, systematic review, vitamins

## Abstract

**Background:**

Associations between vitamin, antioxidant, and fatty acid exposure and coronary heart disease (CHD) risk remain uncertain, and dose–response relationships are poorly defined. This study aimed to evaluate the overall and dose–response associations between dietary and circulating vitamin, antioxidant, and fatty acid levels and CHD risk from observational evidence.

**Methods:**

PubMed, Web of Science, Embase, and the Cochrane Library were searched for studies published up to February 2, 2026 (PROSPERO: CRD420261380823). Eligible observational studies (case–control, cohort, cross-sectional) reporting associations between vitamin, antioxidant, or fatty acid levels and CHD risk were included. A total of 27 studies were analyzed. Categorical (highest vs. lowest) and dose–response analyses were performed, estimating pooled odds ratios (ORs), relative risks (RRs), and hazard ratios (HRs).

**Results:**

Twenty-seven observational studies were included. In categorical analyses, higher circulating 25(OH)D (pooled HR = 0.79, 95% CI = 0.74–0.85; *I*^2^ = 1.4%, *τ*^2^ = 0.0000) and carotenoids (pooled OR = 0.64, 95% CI = 0.46–0.91; *I*^2^ = 0.0%, *τ*^2^ = 0.0000) were inversely associated with CHD risk. In analyses per 1-SD increase in circulating fatty acid levels, linoleic acid was inversely associated with CHD risk (pooled OR = 0.69, 95% CI = 0.57–0.84; *I*^2^ = 0.0%, *τ*^2^ = 0.0000), whereas palmitic acid (pooled OR = 1.52, 95% CI = 1.23–1.89; *I*^2^ = 0.0%, *τ*^2^ = 0.0000) and dihomo-γ-linolenic acid (DGLA; pooled OR = 1.26, 95% CI = 1.05–1.52; *I*^2^ = 0.0%, *τ*^2^ = 0.0000) were positively associated with CHD risk. Several other exposure-specific estimates and most dose–response analyses were supported by only one or two studies and should therefore be interpreted cautiously.

**Conclusion:**

In observational studies, selected dietary and circulating vitamin, antioxidant, and fatty acid exposures were associated with CHD risk. These associations do not establish causality or justify specific supplementation recommendations, and findings based on sparse data require confirmation in additional prospective studies.

## 1 Introduction

Coronary heart disease (CHD) remains one of the leading cardiovascular causes of death and disability worldwide. According to data from the Global Burden of Disease (GBD) study, the global number of CHD cases has reached approximately 240 million, representing a serious threat to human health and quality of life and placing considerable economic and public health pressure on healthcare systems (1, 2). Despite significant progress in clinical interventions targeting traditional cardiovascular risk factors, including hypertension, diabetes, hyperlipidemia, and smoking, the incidence and mortality rates of CHD have remained high (3–6). Consequently, non-traditional intervention strategies, particularly the potential role of dietary and nutritional factors in the primary prevention of CHD, have attracted growing interest in evidence-based medicine and public health. Among various dietary components, vitamins and antioxidants stand as important candidates due to their important role in maintaining vascular homeostasis. As essential micronutrients for normal physiological metabolism and cellular function, certain vitamins (e.g., vitamin C and vitamin E) and their precursors (e.g., β-carotene and selenium) are important constituents of dietary antioxidants (7). These substances may influence key steps in atherosclerosis development by clearing excess free radicals, reducing lipid peroxidation and maintaining endothelial cell integrity (8). Fatty acids were also included because saturated fatty acids, monounsaturated fatty acids, n-3 and n-6 polyunsaturated fatty acids, and trans fatty acids represent biologically distinct lipid exposures. Polyunsaturated fatty acids are particularly relevant to lipid peroxidation and antioxidant defense, providing a biological link between fatty acid profiles and the antioxidant pathways described above (7, 8). Accordingly, dietary intake and circulating levels of individual fatty acid classes were evaluated separately wherever possible. However, observational associations between dietary intake or circulating nutrient levels and CHD do not necessarily imply that supplementation with the same nutrients will prevent CHD; evidence syntheses have not established consistent cardiovascular benefit from antioxidant or vitamin/mineral supplementation (9, 10).

Epidemiological studies have reported associations between vitamin, antioxidant, and fatty acid exposure levels and CHD risk, although the findings have not been consistent. Intervention studies have also produced differing results: a small crocetin trial reported changes in selected antioxidant and inflammatory biomarkers (11), whereas the HOPE trial found no cardiovascular benefit from vitamin E supplementation (12). Such inconsistencies may be partly explained by variation in study size, geographic region, population characteristics and length of follow-up across studies. Furthermore, prior meta-analyses were often confined to a single study design (13, 14) (e.g., cohort studies only) or did not appraise dose–response relationships in a systematic manner, which limited the scope of their conclusions (15). To address this, the present study pools evidence from case–control, cohort and cross-sectional studies within a dose–response meta-analytic framework. This approach allowed us to characterize the nonlinear relationship between vitamin, antioxidant, and fatty acid exposure and CHD risk along two dimensions: dietary intake and circulating biomarker concentrations (blood levels).

## 2 Methods

The conduct and reporting of this systematic review and meta-analysis followed the PRISMA 2020 reporting guidelines.

### 2.1 Search strategy

Four databases were searched systematically: PubMed, Embase, Web of Science and the Cochrane Library. The search covered all records from database inception through February 2, 2026. Full details of the search strategy are provided in Supplementary Table S1.

### 2.2 Inclusion and exclusion criteria

Eligibility criteria were structured using the PECOS framework (Population, Exposure, Comparator, Outcome, and Study design), which was appropriate for the observational exposure studies included in this review (Table 1).

**Table 1:** Eligibility criteria based on the Population, Exposure, Comparator, Outcome, and Study design (PECOS) framework.

PECOS component	Eligibility criteria
Population (P)	General population subjects in epidemiological studies aimed at exploring the association between exposure factors and the risk of coronary heart disease (CHD).
Exposure (E)	Specific intake levels of dietary antioxidants/vitamins/fatty acids, or concentrations of related biomarkers in the subjects’ circulatory system (blood/serum).
Comparator (C)	Defined according to the type of analysis: 1. For categorical overall analyses: The comparator is the lowest dietary exposure or blood concentration category defined in the included studies, or the control population not taking the relevant nutritional supplements. 2. For dose–response analyses: The comparator is the baseline exposure/dose reference group established within each independent epidemiological study
Outcomes (O)	Coronary outcomes reported as coronary heart disease (CHD), ischemic heart disease (IHD), coronary artery disease (CAD), myocardial infarction (MI), acute myocardial infarction (AMI), including nonfatal AMI, or angina. Studies reporting only composite cardiovascular outcomes without a separately extractable coronary endpoint were excluded.
Study design (S)	Observational studies, including cohort studies, case–control studies, and cross-sectional studies.

Studies were eligible if they met the following criteria: (1) The study design is an observational study (including cohort studies, case–control studies, nested case–control studies, or cross-sectional studies); (2) The association between dietary antioxidant, vitamin, or fatty acid intake or circulating concentrations of the corresponding biomarkers and an eligible coronary outcome was evaluated; (3) Multivariable-adjusted effect estimates (e.g., RR, OR, or HR) and 95% confidence intervals (95% CI) were reported; (4) For dose–response analyses, the study must report at least three quantitative exposure levels (including a reference group) along with the number of cases, total sample size, or person-years for each group.

For this review, CHD was used as an umbrella term encompassing the coronary outcomes reported in the original studies, including CHD, ischemic heart disease (IHD), coronary artery disease (CAD), myocardial infarction (MI), acute myocardial infarction (AMI), nonfatal AMI, and angina. CHD, IHD, and CAD were regarded as broader coronary disease outcomes; MI and AMI were regarded as acute coronary events; and angina was regarded as a symptomatic coronary outcome. The exact endpoint terminology used by each original study was retained during data extraction. Studies reporting only a composite cardiovascular outcome without a separately extractable coronary endpoint were excluded.

We excluded the following: (1) Non-observational studies, including reviews, systematic reviews, editorials, expert opinions, conference abstracts, case reports, book chapters, and letters; (2) Animal, cell culture, and *in vitro* studies; (3) studies that did not provide multivariate adjusted effect estimates (OR, RR, or HR) with their 95% confidence intervals (95% CI); (4) studies with no clear exposure classification or dose information.

### 2.3 Data extraction and quality evaluation

Data were extracted by two researchers (Peng Tian and Chang Su) working independently, and any disagreements were settled by consulting a third researcher (Qiuyan Zhang). The following information was collected from each study: first author, publication year, study region, sample size, follow-up duration, sex distribution, the exact coronary endpoint reported by the original study, the specific intake or concentration of the target exposure, and the most fully adjusted effect estimate (RR/HR/OR) with its 95% CI. The “most fully adjusted” estimate refers to the model designated by the original study authors as their maximally adjusted analysis; no fixed minimum set of confounders was required for inclusion, and the specific confounders adjusted for varied across studies according to their design and available data (Supplementary Table S3).

Dose allocation method: In dose–response analysis, the midpoint dose for each exposure dose group is determined as the average of its upper and lower limits; when the exposure dose groups are open-ended intervals, the lowest dose group uses the midpoint of the interval. By contrast, the highest dose group uses 1.2 times the initial value as the average exposure dose (16). To accommodate potential variations in measurement units across studies, all exposure doses were standardized to a common measurement unit before analysis.

Quality assessment: Two reviewers independently assessed cohort and case–control studies using the Newcastle–Ottawa Scale (NOS) (17) and cross-sectional studies using the 11-item Agency for Healthcare Research and Quality (AHRQ) checklist (18). Disagreements were resolved through discussion with a third reviewer. NOS scores of ≥7 and AHRQ scores of ≥8 were classified as high quality. Methodological quality scores were not used as criteria for study inclusion or exclusion. The review protocol was registered in PROSPERO (CRD420261380823) before data collection began, so that the completed review could be compared against the pre-specified plan and unnecessary duplication avoided.

### 2.4 Statistical analysis

For each exposure, quantitative pooling was performed only when at least two comparable estimates were available. Estimates were considered comparable when they used the same effect measure (HR, RR, or OR), assessed the same exposure construct using clinically comparable contrasts, expressed the comparison in the same direction, and evaluated comparable CHD outcomes. Because dietary intake and circulating biomarker levels of the same nutrient reflect different exposure constructs and are subject to different sources of measurement error, they were treated *a priori* as distinct exposures and were never pooled into a single effect estimate; dietary and circulating measures for each nutrient were analyzed in separate, dedicated models throughout this study. HRs, RRs, and ORs were analyzed separately; no HR was combined with an RR or OR, and no RR was combined with an OR in the analyses reported in this study. Although ORs may approximate RRs when the outcome is uncommon, the two measures are not generally equivalent (19, 20). Studies with different observational designs could be included in the same meta-analysis when the above criteria were met. When a publication reported estimates for non-overlapping population strata, these stratum-specific estimates were treated separately. When only one comparable estimate was available, the result was reported as study-specific rather than as pooled evidence.

To assess overall associations between vitamins, antioxidants, and fatty acids with CHD risk, we extracted the contrast between the highest and lowest (reference) exposure categories from all included studies and pooled these estimates. When a ratio measure was reported for the lowest versus highest exposure category, the estimate was inverted to express the highest versus lowest comparison; the corresponding 95% CI was calculated as 1/the original upper limit to 1/the original lower limit. Between-study heterogeneity was examined with Cochran’s *Q* test and the *I*^2^ statistic (21). Studies with *I*^2^ below 50% and *p* > 0.10 were pooled using a fixed-effects model; those with *I*^2^ of 50% or above, or *p* ≤ 0.10, used a random-effects model (22). To look into possible sources of heterogeneity, subgroup analyses were performed according to population characteristics such as age, sex and geographic region. Regional and race/ethnicity subgroup analyses were based on the information reported in the original studies. Strata represented by a single study were reported descriptively rather than treated as pooled subgroup evidence. Regional meta-regression was not performed because too few studies were available for each exposure. Endpoint-based subgroup analyses were considered when at least two independent studies were available within each endpoint category. For mixed-endpoint models in which this condition was not met, an endpoint-based sensitivity analysis was conducted when exclusion of a distinct endpoint still left at least two independent studies for quantitative pooling. Endpoint categories represented by only one remaining independent study were not pooled. Sensitivity analyses were also conducted by sequentially removing each study and recalculating the pooled estimate to assess the stability of results (23). Exploratory funnel plots were generated and are provided in the Supplementary material. Egger’s regression tests were conducted only for the two analyses in which numerical estimation was feasible: the serum 25(OH)D HR model and the overall blood vitamin E OR model. For the remaining analyses, Egger’s tests were not formally estimated because fewer than three independent studies or publications were available for the same exposure and effect measure, or because nutrient-specific estimates were derived from single studies and were not quantitatively pooled. The numerical results of the two estimable tests are reported in the corresponding Results sections and in the captions of Supplementary Figures S5.1, S5.2. Because both analyses included fewer than 10 independent studies or publications, the tests had limited statistical power and were not used to draw formal conclusions regarding publication bias (24).

For studies providing at least three exposure levels, independent dose–response analyses were conducted to examine how CHD risk changed across the exposure range. Restricted cubic spline (RCS) models were fitted to capture potential nonlinear dose–response patterns. Depending on the number of knots used in each nutrient-specific model, three-knot models placed knots at the 10th, 50th, and 90th percentiles; four-knot models at the 5th, 35th, 65th, and 95th percentiles; and five-knot models at the 5th, 27.5th, 50th, 72.5th, and 95th percentiles of the exposure distribution (25). Nonlinearity was assessed with the Wald test; *p* < 0.05 was taken as evidence against a purely linear dose–response pattern (25). For dose–response models based on a single study, between-study variance (*τ*^2^) is not estimable; the goodness-of-fit *p*-value is reported as the indicator of residual heterogeneity for these analyses. Statistical significance of effect estimates was generally indicated as *p* < 0.05 or *p* > 0.05, with the exact value retained when it was at the significance threshold; exact *p*-values were reported for heterogeneity and dose–response model tests, with values below 0.001 reported as *p* < 0.001.

All data processing and statistical analyses were performed in Stata 18 (StataCorp, College Station, TX).

## 3 Results

### 3.1 Literature search results and research characteristics and quality

The study selection process is shown in Figure 1. The database searches identified 65,605 records. After duplicate removal and title and abstract screening, 101 reports were sought for retrieval. Sixteen reports could not be retrieved, leaving 85 full-text reports for eligibility assessment. Following the exclusion of 58 reports after full-text assessment, 27 studies were included in the review, comprising 15 case–control studies (26–40), 11 cohort studies (41–51), and one cross-sectional study (52). The included studies were conducted in North America, Europe, Asia, and Oceania and assessed nutritional exposures through dietary intake, supplement use, or circulating levels.

**Figure 1:**
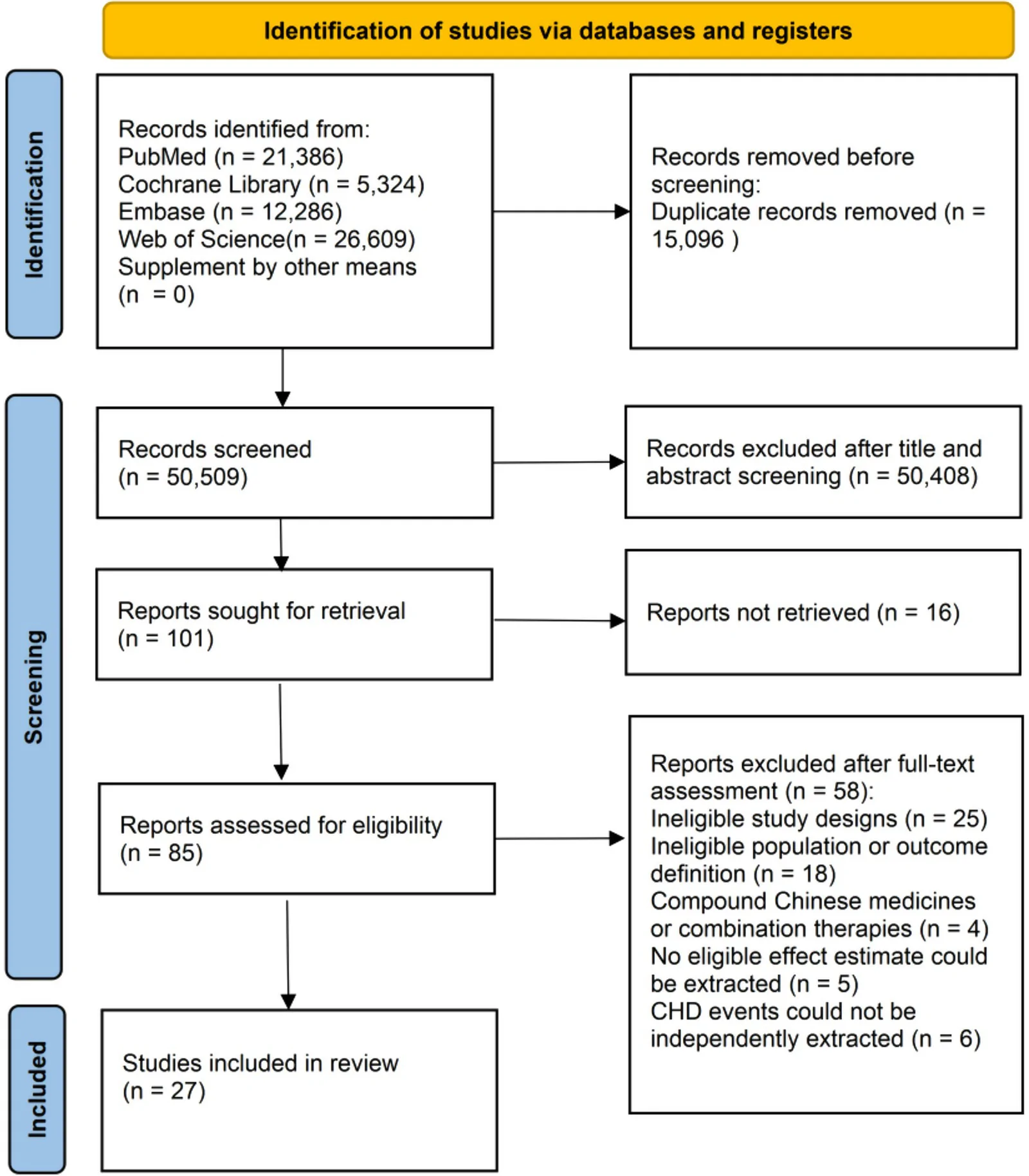
Flow diagram of the literature search and study selection process.

The Newcastle–Ottawa Scale (NOS) was used to assess the cohort and case–control studies. Among these 26 studies, nine received a score of 8 and 17 received a score of 9. The single cross-sectional study was assessed using the AHRQ checklist and received a score of 10. Item-level quality assessments for all included studies are presented in Supplementary Table S2.

### 3.2 Analysis of influencing factors

#### 3.2.1 Intake of multivitamins and CHD risk

The analysis included two cohort studies, involving 55,723 participants. The pooled estimate showed no significant association between multivitamins intake and the risk of CHD (combined HR = 0.95, 95% CI = 0.83–1.10, *p* > 0.05). Heterogeneity was moderate among the included studies (*I*^2^ = 62.1%, *τ*^2^ = 0.0066, *p* = 0.104) (Supplementary Figure S1.1).

Because sex-specific estimates were available and sex composition could have contributed to heterogeneity, we conducted subgroup analyses by sex. The male subgroup showed a borderline inverse association (HR = 0.89, 95% CI = 0.79–1.00, *p* = 0.050), whereas no statistically significant association was observed in the female subgroup (HR = 1.03, 95% CI = 0.90–1.17, *p* > 0.05) (Supplementary Figure S1.2).

#### 3.2.2 Vitamin D intake and CHD risk

The analysis included two independent cohort studies. Because the studies differed substantially in terms of exposure assessment methods (qualitative supplement use versus quantitative dietary intake) and reported effect size measures (HR and RR), to minimize clinical heterogeneity, we did not combine their data across studies but instead constructed separate forest plots for visual comparison.

In the cohort study assessing vitamin D supplement use qualitatively, neither the overall estimate (HR = 1.00, 95% CI = 0.89–1.13, *p* > 0.05) nor the male- and female-specific estimates showed a statistically significant association with CHD risk (males: HR = 0.95, 95% CI = 0.80–1.13; females: HR = 1.05, 95% CI = 0.89–1.25; both *p* > 0.05) (Supplementary Figure S1.3).

In the cohort study assessing quantitative dietary vitamin D intake, the highest versus lowest intake comparison was not statistically significantly associated with CHD risk overall (RR = 0.95, 95% CI = 0.78–1.15, *p* > 0.05), in males (RR = 0.86, 95% CI = 0.72–1.02, *p* > 0.05), or in females (RR = 1.05, 95% CI = 0.87–1.26, *p* > 0.05) (Supplementary Figure S1.4).

#### 3.2.3 Serum 25(OH)D levels and CHD risk

The analysis included five studies involving 80,947 participants, and the effect measures were analyzed separately. The HR model shown in Figure 2A included two prospective cohort studies and one nested case–control study. Although the study designs differed, all studies reported adjusted HRs for the association between serum 25(OH)D levels and CHD risk, and their exposure measures and CHD outcomes were considered comparable. Therefore, these studies were included in the same HR meta-analysis. Higher serum 25(OH)D levels were significantly associated with a lower risk of CHD (pooled HR = 0.79; 95% CI = 0.74–0.85; *p* < 0.05), with low statistical heterogeneity (*I*^2^ = 1.4%, *τ*^2^ = 0.0000, *p* = 0.398) (Figure 2A). Because the HR model combined broader CHD/IHD outcomes with one MI-specific outcome, we conducted an endpoint-based sensitivity analysis excluding the MI-specific study by Acharya et al. The inverse association remained similar after exclusion of this study (pooled HR = 0.80, 95% CI = 0.74–0.86; *I*^2^ = 19.0%, *p* = 0.295). However, the remaining four population-specific estimates were derived from only two publications, and this sensitivity result should therefore be interpreted cautiously. Egger’s regression test showed no statistical evidence of funnel plot asymmetry (bias coefficient = 0.875, 95% CI = −2.482 to 4.232, *p* = 0.468). However, because the model included only three publications contributing five non-overlapping estimates, the test had limited statistical power and the result was considered exploratory only (Supplementary Figure S5.1).

**Figure 2:**
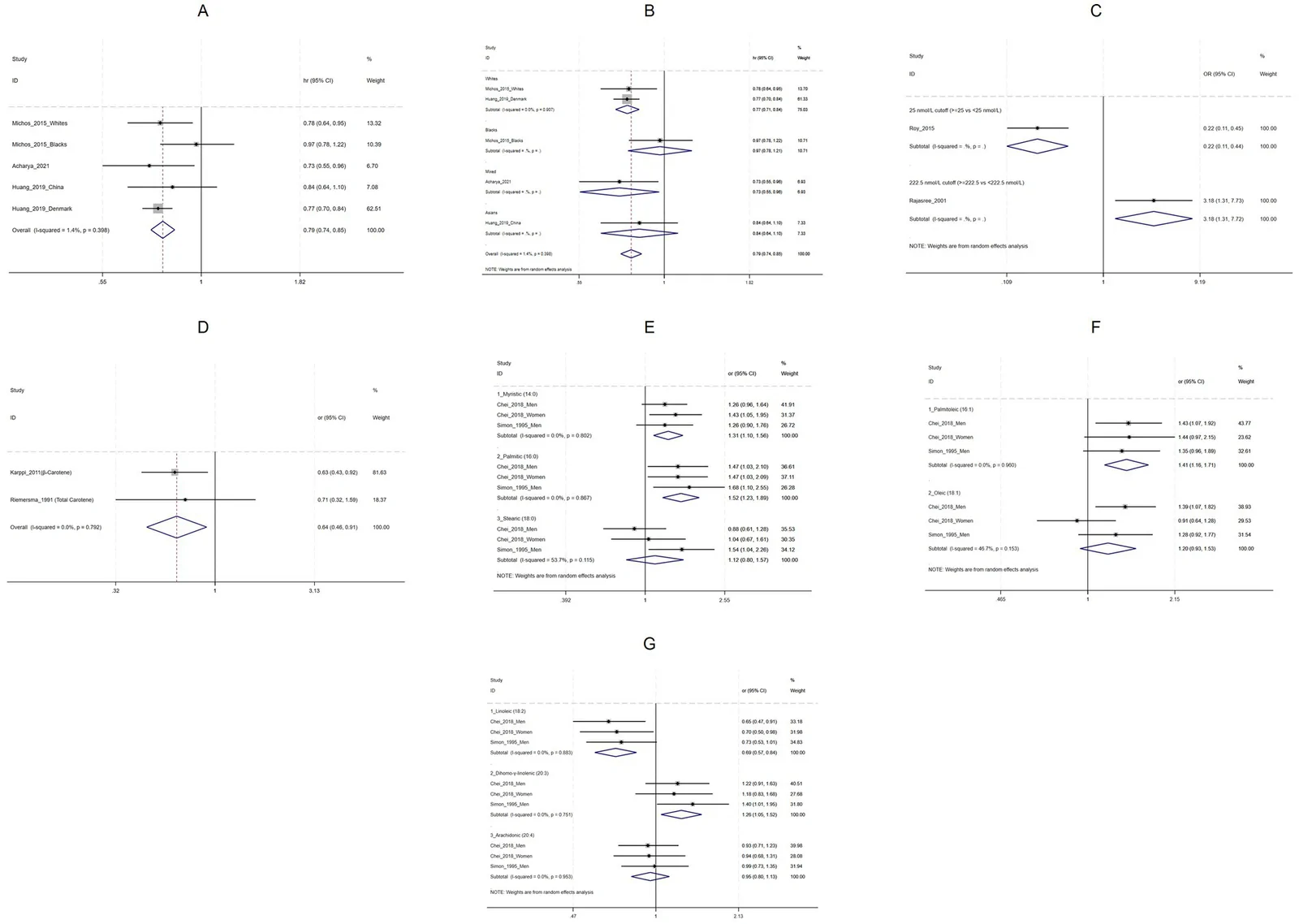
Forest plots of the association between various vitamin, antioxidant, and fatty acid levels and the risk of coronary heart disease (CHD). **(A)** Forest plot of the association between serum 25(OH)D levels and the risk of CHD (effect size: HR); **(B)** Forest plot of the association between serum 25(OH)D levels and the risk of CHD in ethnicity subgroups (effect size: HR); **(C)** Forest plot of the association between serum 25(OH)D levels and CHD risk using study-specific concentration cutoffs (≥25 vs. <25 nmol/L and ≥222.5 vs. <222.5 nmol/L); **(D)** Forest plot of the association between blood carotene levels and the risk of CHD; **(E)** Forest plot of the association between blood saturated fatty acid (SFA) levels and the risk of CHD in subgroups; **(F)** Forest plot of the association between blood monounsaturated fatty acid (MUFA) levels and the risk of CHD in subgroups; **(G)** Forest plot of the association between blood n-6 polyunsaturated fatty acid (n-6 PUFA) levels and the risk of CHD in subgroups.

We stratified the association between circulating 25(OH)D and CHD risk according to the racial or ethnic information reported in the original studies (Figure 2B). The White subgroup included two studies and yielded a pooled HR of 0.77 (95% CI = 0.71–0.84). The mixed, Asian, and Black subgroups were each represented by a single study, with HRs of 0.73 (95% CI = 0.55–0.96), 0.84 (95% CI = 0.64–1.10), and 0.97 (95% CI = 0.78–1.21), respectively. The confidence intervals crossed 1.0 in the Asian and Black subgroups. Although the point estimates varied across subgroups, overall heterogeneity was low (*I*^2^ = 1.4%, *τ*^2^ = 0.0000, *p* = 0.398), and most subgroups were represented by only one study. The available data therefore do not establish whether the association between circulating 25(OH)D and CHD risk differs by race or ethnicity.

Furthermore, using ORs reported by two case–control studies, we examined the associations at two study-specific serum 25(OH)D concentration cutoffs. The associations were in opposite directions, resulting in substantial between-study heterogeneity (*I*^2^ = 95.3%, *p* < 0.001); the two studies were therefore presented separately rather than pooled (Figure 2C).

One study defined severe deficiency as serum 25(OH)D < 10 ng/mL (<25 nmol/L) (36). After harmonizing the comparison direction, the OR for participants without severe deficiency (≥25 nmol/L) versus those with severe deficiency (<25 nmol/L) was 0.22 (95% CI = 0.11–0.45, *p* < 0.05). The other study used a cutoff of 222.5 nmol/L (32); this was a case–control study conducted in a male Indian population (45–65 years) comprising 213 participants, including 143 CHD cases. The OR for ≥222.5 versus <222.5 nmol/L was 3.18 (95% CI = 1.31–7.73, *p* < 0.05), based on this single small study.

#### 3.2.4 Blood vitamin E levels and CHD risk

The pooled estimate showed no statistically significant association between blood vitamin E levels and CHD risk (pooled OR = 1.00, 95% CI = 0.54–1.85, *p* > 0.05), with substantial between-study heterogeneity (*I*^2^ = 71.2%, *τ*^2^ = 0.2036, *p* = 0.031) (Supplementary Figure S1.5). Egger’s regression test showed no statistical evidence of funnel plot asymmetry (bias coefficient = −4.553, 95% CI = −31.126 to 22.019, *p* = 0.274). However, because only three independent studies were included, the test had very limited statistical power, as reflected by the wide confidence interval, and the result was considered exploratory only (Supplementary Figure S5.2). Because Riemersma et al. assessed questionnaire-defined angina, whereas Hense et al. and Karppi et al. assessed MI or AMI, we conducted an endpoint-based sensitivity analysis excluding the angina study. After excluding Riemersma et al., between-study heterogeneity decreased substantially (*I*^2^ = 0.0%, *p* = 0.895), while the pooled association remained statistically non-significant (pooled OR = 1.35, 95% CI = 0.99–1.85; *p* > 0.05) (Supplementary Figure S2.1). Although the point estimate changed after exclusion, its 95% CI still included 1.0. This finding indicates that differences in coronary endpoint definitions influenced the pooled estimate, but it does not establish an association between blood vitamin E levels and CHD risk.

When the reported vitamin E concentration categories were stratified at 20 μmol/L, no statistically significant association was observed in either the <20 μmol/L subgroup (pooled OR = 0.75, 95% CI = 0.22–2.61, *p* > 0.05; *I*^2^ = 84.3%, *τ*^2^ = 0.6899, P for heterogeneity = 0.012) or the >20 μmol/L subgroup (pooled OR = 1.16, 95% CI = 0.88–1.52, *p* > 0.05; *I*^2^ = 0.0%, *τ*^2^ = 0.0000, P for heterogeneity = 0.609) (Supplementary Figure S1.6). Substantial heterogeneity was observed in the <20 μmol/L subgroup, whereas no between-estimate heterogeneity was detected in the >20 μmol/L subgroup. Because only a small number of study-specific estimates contributed to each subgroup, these exploratory results should be interpreted cautiously.

#### 3.2.5 Blood carotenoid levels and CHD risk

The analysis included two studies involving 1,535 participants. Based on the biological comparability of the exposure measures, total circulating carotenoids and β-carotene were grouped as “circulating carotenoids” for pooled evaluation. Limited pooled evidence from these two studies suggested that higher circulating carotenoid levels were associated with a lower risk of CHD (pooled OR = 0.64, 95% CI = 0.46–0.91, *p* < 0.05) (Figure 2D). No statistical heterogeneity was detected (*I*^2^ = 0.0%, *τ*^2^ = 0.0000, *p* = 0.792); however, the small number of studies precludes firm conclusions regarding the consistency of this association.

#### 3.2.6 Blood saturated fatty acids (SFA) and CHD risk

The analysis included two independent studies. Considering the potential heterogeneous biological effects of saturated fatty acids with different carbon chain lengths, we conducted separate effect size pooled analyses for the three major subtypes of SFA: myristic acid (C14: 0), palmitic acid (C16: 0), and stearic acid (C18: 0). All effect sizes were quantitatively assessed using a continuous variable model based on an increase of one standard deviation (Per 1-SD) in blood fatty acid concentrations.

Limited pooled evidence from two studies suggested that the associations between circulating saturated fatty acids and CHD risk differed by subtype. Each 1-SD increase in circulating myristic acid (C14:0) was associated with a higher risk of CHD (pooled OR = 1.31, 95% CI = 1.10–1.56, *p* < 0.05), with no statistical heterogeneity detected (*I*^2^ = 0.0%, *τ*^2^ = 0.0000, *p* = 0.802). Similarly, each 1-SD increase in circulating palmitic acid (C16:0) was associated with a higher risk of CHD (pooled OR = 1.52, 95% CI = 1.23–1.89, *p* < 0.05), with no statistical heterogeneity detected (*I*^2^ = 0.0%, *τ*^2^ = 0.0000, *p* = 0.867). In contrast, no statistically significant association was observed for circulating stearic acid (C18:0) (pooled OR = 1.12, 95% CI = 0.80–1.57, *p* > 0.05), and moderate between-study heterogeneity was present (*I*^2^ = 53.7%, *τ*^2^ = 0.0479, *p* = 0.115) (Figure 2E). Sensitivity analysis suggested that the study by Simon et al. contributed to this heterogeneity. After excluding this study, the pooled point estimate shifted to the left, but the 95% CI continued to cross the null value of 1.0; therefore, the statistical inference remained unchanged (Supplementary Figure S2.2). Nevertheless, the change in the point estimate indicates that the estimated magnitude of the association was sensitive to the included data. Simon et al. investigated a high-risk male population in the United States and measured specific cholesterol ester fractions, whereas the remaining data were based on total serum fatty acid measurements in a general Japanese community population. Differences in population characteristics and biomarker measurements may have contributed to the observed heterogeneity. Because each subtype-specific pooled estimate was based on only two studies, both the association estimates and the heterogeneity statistics should be interpreted cautiously.

#### 3.2.7 Monounsaturated fatty acids (MUFA) and CHD risk

The analysis included two independent studies. Given the potential heterogeneous biological effects of monounsaturated fatty acids with varying carbon chain lengths and double bond positions, we conducted separate effect size pooled analyses for the two major MUFA subtypes: palmitoleic acid (C16: 1) and oleic acid (C18: 1). All effect sizes were quantitatively assessed using a continuous variable model based on an increase of one standard deviation (Per 1-SD) in blood fatty acid concentrations.

Limited pooled evidence from two studies suggested that each 1-SD increase in circulating palmitoleic acid (C16:1) was associated with a higher risk of CHD (pooled OR = 1.41, 95% CI = 1.16–1.71, *p* < 0.05). No statistical heterogeneity was detected for this estimate (*I*^2^ = 0.0%, *τ*^2^ = 0.0000, *p* = 0.960). In contrast, no statistically significant association was observed between circulating oleic acid (C18:1) and CHD risk (pooled OR = 1.20, 95% CI = 0.93–1.53, *p* > 0.05), with some between-study heterogeneity (*I*^2^ = 46.7%, *τ*^2^ = 0.0222, *p* = 0.153) (Figure 2F). Because both pooled estimates were based on only two studies, the associations and heterogeneity statistics should be interpreted cautiously.

#### 3.2.8 Blood N-3 polyunsaturated fatty acids (n-3 PUFA) and CHD risk

The analysis included two independent studies. We conducted a pooled effect analysis for the four major subtypes within the blood n-3 polyunsaturated fatty acid (n-3 PUFA) family, alpha-linolenic acid (C18: 3n3), eicosapentaenoic acid (EPA, C20: 5), docosapentaenoic acid (DPA, C22: 5), and docosahexaenoic acid (DHA, C22: 6). All effect sizes were quantitatively assessed using a continuous variable model based on an increase of one standard deviation (Per 1-SD) in exposure concentration.

No statistically significant associations were observed for circulating α-linolenic acid (pooled OR = 0.84, 95% CI = 0.70–1.01, *p* > 0.05; *I*^2^ = 0.0%, *τ*^2^ = 0.0000, *p* = 0.839), EPA (pooled OR = 1.03, 95% CI = 0.82–1.30, *p* > 0.05; *I*^2^ = 36.5%, *τ*^2^ = 0.0157, *p* = 0.207), DHA (pooled OR = 0.81, 95% CI = 0.63–1.05, *p* > 0.05; *I*^2^ = 44.5%, *τ*^2^ = 0.0230, *p* = 0.165), or DPA (pooled OR = 0.98, 95% CI = 0.62–1.56, *p* > 0.05; *I*^2^ = 79.4%, *τ*^2^ = 0.1326, *p* = 0.008) (Supplementary Figure S1.7). Sensitivity analysis indicated that the Simon et al. dataset contributed substantially to the heterogeneity for DPA. After its exclusion, the point estimate shifted markedly to the right, but the confidence interval continued to include 1.0 (Supplementary Figure S2.3). Thus, although the statistical conclusion was unchanged, the magnitude of the pooled DPA estimate was sensitive to the included data and should be interpreted cautiously.

#### 3.2.9 Blood N-6 polyunsaturated fatty acids (n-6 PUFA) and CHD risk

The analysis included two independent studies. We conducted independent effect size combination analyses for linoleic acid (C18: 2), dihomo-γ-linolenic acid (DGLA, C20:3), and arachidonic acid (C20: 4) within the n-6 polyunsaturated fatty acid (PUFA) family in blood. All effect sizes were assessed using a continuous variable model based on an increase of one standard deviation (Per 1-SD) in blood exposure concentration.

Limited pooled evidence from two studies suggested that each 1-SD increase in circulating linoleic acid was associated with a lower risk of CHD (pooled OR = 0.69, 95% CI = 0.57–0.84, *p* < 0.05), whereas each 1-SD increase in circulating dihomo-γ-linolenic acid (DGLA) was associated with a higher risk (pooled OR = 1.26, 95% CI = 1.05–1.52, *p* < 0.05). No statistically significant association was observed for circulating arachidonic acid (pooled OR = 0.95, 95% CI = 0.80–1.13, *p* > 0.05). No statistical heterogeneity was detected for linoleic acid (*I*^2^ = 0.0%, *τ*^2^ = 0.0000, *p* = 0.883), DGLA (*I*^2^ = 0.0%, *τ*^2^ = 0.0000, *p* = 0.751), or arachidonic acid (*I*^2^ = 0.0%, *τ*^2^ = 0.0000, *p* = 0.953) (Figure 2G). However, because each pooled estimate was based on only two studies, the absence of detected heterogeneity should not be interpreted as establishing consistency, and the associations should be interpreted cautiously.

#### 3.2.10 Blood vitamin B complex (B6, B12, folate) and CHD risk

Two independent studies evaluated circulating vitamin B6, vitamin B12, and folate in relation to CHD risk. As each biomarker was examined in only one study, the estimates were not pooled. For vitamin B6, one retrospective case–control study conducted in the Netherlands (Kok et al.) reported the effect estimate for the lowest versus highest quartile of PLP; after harmonizing the comparison direction, the OR for acute myocardial infarction comparing the highest with the lowest quartile was 0.19 (95% CI = 0.05–0.71, *p* < 0.05). Higher circulating folate was also associated with a lower risk of CHD (OR = 0.40, 95% CI = 0.30–0.90, *p* < 0.05), whereas vitamin B12 showed no significant association (OR = 1.20, 95% CI = 0.50–2.50, *p* > 0.05) (Supplementary Figure S1.8).

#### 3.2.11 Dietary antioxidant intake levels and CHD risk

The analysis included two independent studies (including one cross-sectional study and one case–control study), each evaluating the association between intake levels of vitamin E, β-carotene, lycopene, and retinol with the risk of coronary heart disease (CHD). Since each specific dietary antioxidant indicator was reported independently in individual original studies, the statistical criteria for quantitative combination of effect sizes were not met. Therefore, the findings were summarized study by study. The highest category of dietary vitamin E intake was not significantly associated with CHD risk (OR = 0.81, 95% CI = 0.64–1.03, *p* > 0.05). Higher dietary β-carotene intake was inversely associated with CHD risk (OR = 0.71, 95% CI = 0.51–0.98, *p* < 0.05). Dietary lycopene (OR = 1.19, 95% CI = 0.82–1.70, *p* > 0.05) and retinol (OR = 1.00, 95% CI = 0.70–1.42, *p* > 0.05) were not significantly associated with CHD risk (Supplementary Figure S1.9).

#### 3.2.12 Blood antioxidant concentration and CHD risk

The analysis included two independent studies (including one cohort study and one case–control study), each evaluating the association between serum concentrations of vitamin C, vitamin E, total carotenoids, β-carotene, lycopene, and retinol with the risk of coronary heart disease (CHD). Since each specific biomarker was reported independently only in its original study and does not currently meet the criteria for quantitative combination, descriptive inferences were drawn regarding the independent associations of each biomarker.

In case–control studies, the highest percentile of blood vitamin E concentration showed a statistically significant association with a reduced risk of CHD (OR = 0.37, 95% CI = 0.15–0.93, *p* < 0.05). In contrast, the point estimates for blood vitamin C (OR = 0.61, 95% CI = 0.29–1.32, *p* > 0.05) and total carotene (OR = 0.71, 95% CI = 0.32–1.59, *p* > 0.05), measured in the same population, were below 1.0, but neither association was statistically significant.

In the cohort study, higher circulating β-carotene (OR = 0.63, 95% CI = 0.43–0.92, *p* < 0.05) and lycopene (OR = 0.65, 95% CI = 0.43–0.95, *p* < 0.05) levels were inversely associated with CHD risk, whereas serum retinol levels showed no statistically significant association (OR = 0.86, 95% CI = 0.60–1.23, *p* > 0.05) (Supplementary Figure S1.10).

### 3.3 Analysis of dose–response results

#### 3.3.1 Dietary vitamin C intake and CHD risk

Only one cohort study was included, and the analysis was restricted to participants who were not taking vitamin supplements at baseline. The fitted curve and category-specific estimates varied across the observed range of dietary vitamin C intake (Figure 3). In the original categorical data, the RR point estimate was 0.92 (95% CI = 0.79–1.07) at 84 mg/day and 1.23 (95% CI = 1.04–1.45) at 152 mg/day. The overall dose–response model was statistically significant (*p* = 0.0031), and the test for nonlinearity was also statistically significant (*p* = 0.0057), providing evidence of a nonlinear dose–response pattern. The goodness-of-fit test was not statistically significant (*p* = 0.2683), indicating no statistical evidence of residual lack of fit across the exposure categories. Nevertheless, because this analysis was derived from a single cohort study with only five exposure categories, the fitted pattern should be considered exploratory and should not be interpreted as evidence that moderate vitamin C intake reduces CHD risk or that higher intake reverses the direction of the association. These data cannot establish an optimal intake range or a risk threshold.

**Figure 3:**
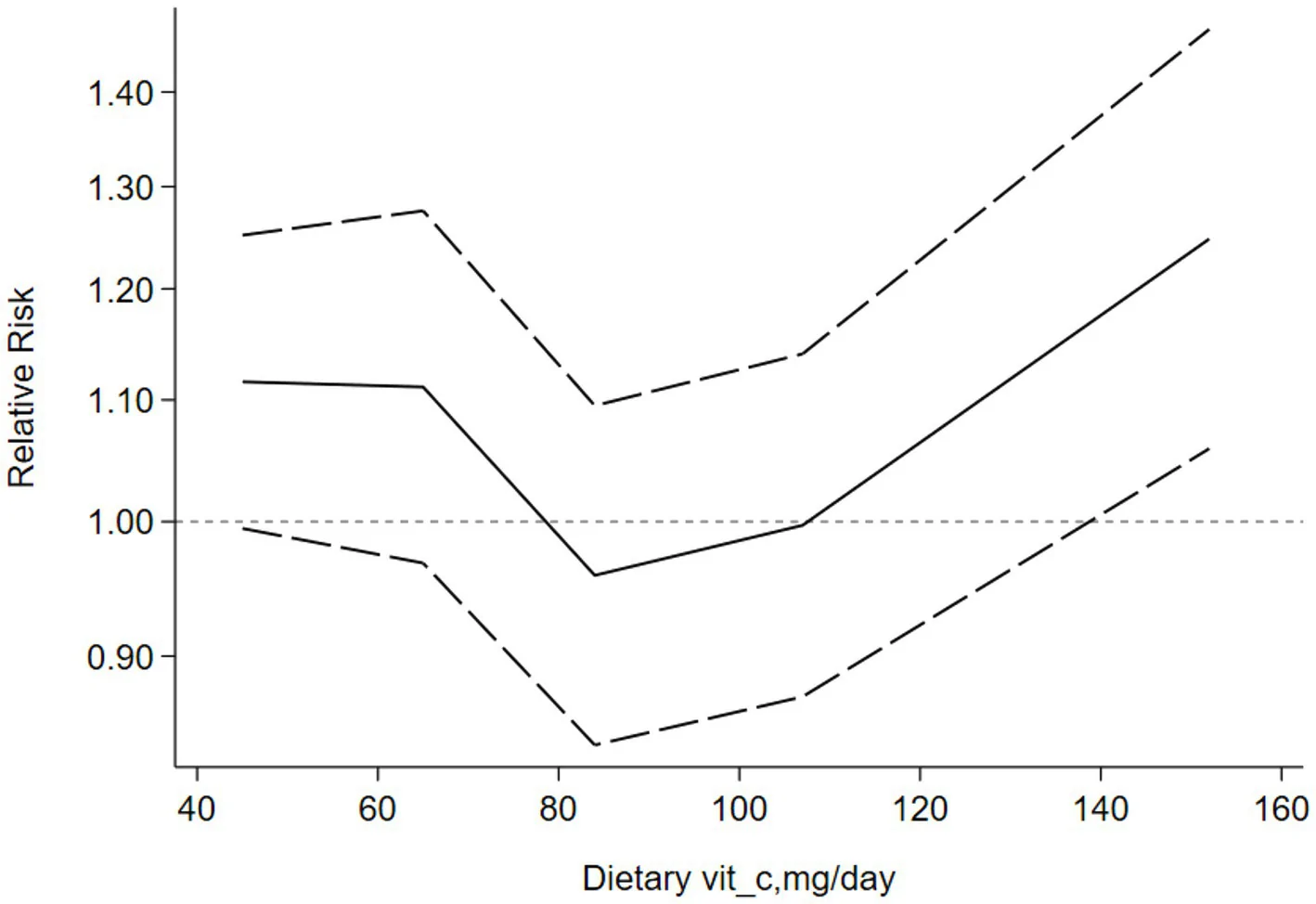
Five-knot restricted cubic spline dose–response curve of the association between dietary vitamin C intake and CHD risk. Knots were placed at the 5th, 27.5th, 50th, 72.5th, and 95th percentiles of the exposure distribution.

#### 3.3.2 Dietary vitamin E intake and CHD risk

Data from two cohort studies were combined. In the original categorical data, the RR point estimate at 8.64 mg/day was 0.79 (95% CI = 0.54–1.15) (Supplementary Figure S3.1). The overall dose–response association did not reach the conventional threshold for statistical significance (*p* = 0.0510), and the test for nonlinearity was also not statistically significant (*p* = 0.1192). The goodness-of-fit test showed no statistical evidence of residual lack of fit (*p* = 0.5514). Because a fixed-effects generalized least-squares model was used, *τ*^2^ was not a model parameter. Therefore, the limited pooled evidence from two cohort studies did not establish a statistically significant dose–response association between dietary vitamin E intake and CHD risk.

#### 3.3.3 Dietary lycopene intake and CHD risk

Only one cohort study provided category-specific dose–response data. In the original categorical data, the RR point estimate at a dietary lycopene intake of 1.33 mg/day was 1.08 (95% CI = 0.95–1.24), while the estimates at higher intake levels remained close to the null value (Supplementary Figure S3.2). The five-knot restricted cubic spline model showed no statistically significant overall dose–response association (*p* = 0.5243) and no evidence of nonlinearity (*p* = 0.4240). The goodness-of-fit test showed no statistical evidence of residual lack of fit (*p* = 0.7904). Therefore, the available single-study evidence did not establish a dose–response association between dietary lycopene intake and CHD risk.

#### 3.3.4 Dietary carotene intake and CHD risk

To investigate the association between dietary carotene intake and CHD risk, we examined two distinct exposures—total carotene and β-carotene—and analyzed them separately according to the study designs and effect measures (RR or OR) reported in the original studies.

In one cohort study of dietary total carotene intake, the fitted RR curve showed a slight downward pattern across the observed exposure range, but the confidence interval included the null value throughout the curve (Supplementary Figure S3.3). In the original categorical data, the RR point estimate at the highest reported intake of 1.38 mg/day was 0.91 (95% CI = 0.78–1.06). Neither the overall dose–response association (*p* = 0.5499) nor the test for nonlinearity (*p* = 0.7942) was statistically significant. The goodness-of-fit test showed no statistical evidence of residual lack of fit (*p* = 0.3709). Therefore, this single-study analysis did not establish a dose–response association between dietary total carotene intake and CHD risk.

In the cohort study reporting RRs for dietary β-carotene, the fitted curve showed a slight downward pattern at higher intake levels, but the confidence interval included the null value across the observed range (Supplementary Figure S3.4). In the original categorical data, the RR point estimate at the highest reported intake of 5.23 mg/day was 0.92 (95% CI = 0.79–1.06). The overall dose–response association was not statistically significant (*p* = 0.6859), and there was no evidence of nonlinearity (*p* = 0.9208). The goodness-of-fit test showed no statistical evidence of residual lack of fit (*p* = 0.3177). Therefore, this single-study RR analysis did not establish a dose–response association between dietary β-carotene intake and CHD risk.

In the case–control study reporting ORs, the fitted curve showed a decrease in CHD risk with increasing dietary β-carotene intake (Supplementary Figure S3.5). In the original categorical data, the OR at the highest reported intake of 5.50 mg/day was 0.50 (95% CI = 0.30–0.80). The overall dose–response association was statistically significant (*p* = 0.0100), whereas the test for nonlinearity was not statistically significant (*p* = 0.8757), indicating no evidence of departure from an approximately linear association. The goodness-of-fit test showed no statistical evidence of residual lack of fit (*p* = 0.6827). However, because this finding was derived from a single case–control study, it should be interpreted cautiously and does not establish a causal protective effect of dietary β-carotene.

This divergence arising from the study design may reflect the inherent susceptibility of case–control studies to retrospective dietary assessment bias or substantial variation in baseline antioxidant status across populations. Overall, the current level of evidence is insufficient to demonstrate that β-carotene independently reduces the risk of CHD.

#### 3.3.5 Dietary retinol intake and CHD risk

Only one case–control study provided category-specific dose–response data. The fitted curve showed minor fluctuations around the null value across the observed range of dietary retinol intake, and the confidence interval included the null value throughout the curve (Supplementary Figure S3.6). In the original categorical data, the OR at the highest reported intake of 2.88 mg/day was 0.90 (95% CI = 0.60–1.30). The overall dose–response association was not statistically significant (*p* = 0.6428), and there was no evidence of nonlinearity (*p* = 0.6306). The goodness-of-fit test showed no statistical evidence of residual lack of fit (*p* = 0.8598). Therefore, this single-study analysis did not establish a dose–response association between dietary retinol intake and CHD risk.

#### 3.3.6 Dietary omega-3 intake and CHD risk

Only one cohort study provided category-specific dose–response data on dietary omega-3 fatty acid intake and CHD risk. In the original categorical data, the RR point estimate was 0.94 (95% CI = 0.80–1.12) at 0.3 g/day and 1.15 (95% CI = 0.97–1.35) at 0.8 g/day. Consistent with these category-specific estimates, the fitted curve showed a slight decrease at lower intake levels followed by an increase at higher intake levels (Supplementary Figure S3.7). However, neither the overall dose–response association (*p* = 0.1263) nor the test for nonlinearity (*p* = 0.3293) was statistically significant. The goodness-of-fit test showed no statistical evidence of residual lack of fit (*p* = 0.5447). Therefore, this single-study analysis did not establish a dose–response association between dietary omega-3 fatty acid intake and CHD risk, and the observed change in the direction of the point estimates should not be interpreted as a risk threshold or reversal of association.

#### 3.3.7 Dietary intake of saturated fatty acids and its relationship with CHD risk

Only one cohort study provided category-specific dose–response data on dietary saturated fatty acid intake and CHD risk. In the original categorical data, the RR at an intake of 50.3 g/day was 0.82 (95% CI = 0.69–0.97). The fitted curve showed a modest decrease followed by a slight increase at higher intake levels, but the confidence interval included the null value throughout the curve (Supplementary Figure S3.8). Neither the overall dose–response association (*p* = 0.2441) nor the test for nonlinearity (*p* = 0.5560) was statistically significant. The goodness-of-fit test showed no statistical evidence of residual lack of fit (*p* = 0.1144). Therefore, the category-specific estimate at 50.3 g/day should not be interpreted as establishing a protective intake level, and this single-study analysis did not establish a dose–response association between dietary saturated fatty acid intake and CHD risk.

#### 3.3.8 Dietary intake of trans fatty acids and its relationship with CHD risk

Only one cohort study provided category-specific dose–response data on dietary trans fatty acid intake and CHD risk. In the original categorical data, the RR point estimates fluctuated modestly across intake levels, with the lowest point estimate observed at 2.0 g/day (RR = 0.97, 95% CI = 0.82–1.16) and an RR of 1.14 (95% CI = 0.96–1.35) at the highest reported intake of 6.2 g/day (Supplementary Figure S3.9). Neither the overall dose–response association (*p* = 0.4035) nor the test for nonlinearity (*p* = 0.7441) was statistically significant. The goodness-of-fit test showed no statistical evidence of residual lack of fit (*p* = 0.1880). Therefore, this single-study analysis did not establish a dose–response association between dietary trans fatty acid intake and CHD risk, and the fluctuations in the category-specific point estimates should not be interpreted as a risk threshold or reversal of association.

#### 3.3.9 Serum 25(OH)D concentration and CHD risk

The analysis included three studies. Since the original studies reported different effect size measures for the relationship between serum 25(OH)D concentrations and the risk of CHD, we conducted independent dose–response analyses separately for studies using hazard ratios (HR) and odds ratios (OR) as the effect sizes.

One HR-based study showed a decreasing point-estimate pattern with increasing serum 25(OH)D concentrations (Supplementary Figure S3.10), but neither the overall dose–response association (*p* = 0.117) nor the test for nonlinearity (*p* = 0.082) was statistically significant. Residual heterogeneity could not be assessed because too few dose categories were available. A pooled analysis of two studies using OR as the effect size revealed minimal fluctuations in the curves visually but no statistically significant nonlinear dose–response relationship (*p* = 0.446) (Supplementary Figure S3.11). The model goodness-of-fit test indicated no significant residual heterogeneity (*p* = 0.620), and the overall dose–response model association was not statistically significant (*p* = 0.293). Overall, the available evidence, including one HR study and two OR studies, did not support a statistically significant dose–response association between serum 25(OH)D concentrations and CHD risk.

#### 3.3.10 Blood α-tocopherol concentration and CHD risk

Only one nested case–control study provided category-specific dose–response data on blood α-tocopherol concentrations and CHD risk. The fitted OR curve fluctuated above the null value and increased at the upper end of the observed concentration range, but its confidence interval included the null value throughout the curve (Supplementary Figure S3.12). In the original categorical data, the OR at the highest reported concentration of 44.2 μmol/L was 2.09 (95% CI = 0.96–4.52). Neither the overall dose–response association (*p* = 0.2197) nor the test for nonlinearity (*p* = 0.5641) was statistically significant. The goodness-of-fit test showed no statistical evidence of residual lack of fit (*p* = 0.7059). Therefore, this single-study analysis did not establish the direction or magnitude of the association between blood α-tocopherol concentrations and CHD risk.

#### 3.3.11 Blood vitamin B6 (PLP) concentration and CHD risk

Regarding the levels of pyridoxal phosphate (PLP), the primary active form of vitamin B6 in blood, a single nested case–control study has provided limited evidence. Analysis of the dose–response fit curve reveals that the risk of coronary heart disease (CHD) generally decreases with increasing blood PLP concentrations; although a modest rebound in risk effect is observed at an exposure concentration of 55.2 nmol/L (RR = 0.66), the risk further declines with higher concentrations (Supplementary Figure S3.13). Statistical tests indicate that the overall association of this dose–response relationship is statistically significant (*p* = 0.002), although the nonlinearity test did not reach statistical significance (*p* = 0.080). The curve showed an overall inverse pattern, but residual heterogeneity could not be assessed because the original study included few dose categories. As the analysis was based on a single nested case–control study, it cannot determine the true magnitude of the association or support a causal interpretation.

#### 3.3.12 Plasma ubiquinol concentration and CHD risk

Only one nested case–control study provided category-specific dose–response data on plasma ubiquinol concentrations and CHD risk. Using 0.44 μmol/L as the reference concentration, the OR point estimate in the original categorical data was 0.61 (95% CI = 0.33–1.13) at 0.60 μmol/L and subsequently increased to 1.22 (95% CI = 0.62–2.40) in the highest concentration category of 1.14 μmol/L (Supplementary Figure S3.14). However, neither the overall dose–response association (*p* = 0.1816) nor the test for nonlinearity (*p* = 0.0889) was statistically significant. The goodness-of-fit test showed no statistical evidence of residual lack of fit (*p* = 0.8126). Therefore, this single-study analysis did not establish a dose–response association between plasma ubiquinol concentrations and CHD risk, and the change in the direction of the point estimates should not be interpreted as a reversal of a protective effect.

## 4 Discussion

### 4.1 Main research findings

This study included 27 observational studies evaluating dietary and circulating vitamins, antioxidants, and fatty acids in relation to CHD risk. Pooled analyses showed that higher circulating 25(OH)D, carotenoid, and linoleic acid levels were associated with lower CHD risk, whereas myristic acid, palmitic acid, palmitoleic acid, and DGLA were associated with higher risk. Pooled analyses of multivitamin use, circulating vitamin E, stearic acid, oleic acid, and n-3 PUFAs showed no clear associations, and several analyses exhibited moderate or substantial between-study heterogeneity. For circulating B vitamins and several dietary or circulating antioxidants, only one study was available for each exposure and the estimates were not pooled. These study-specific findings provide less certain evidence than the pooled estimates and require confirmation in independent populations. Most nutrient-specific dose–response analyses were also based on a single study; the resulting curves therefore describe exposure-risk patterns within the corresponding study rather than established optimal ranges or risk thresholds.

### 4.2 Comparison with other studies

Previous meta-analyses have mainly focused on dietary or supplemental intake of specific vitamins. Ye et al. (13) included 15 cohort studies evaluating the associations of vitamin C, vitamin E, and β-carotene intake with CHD risk. In their linear dose–response analyses, the pooled RR was 1.01 (95% CI = 0.99–1.02) for each 30 mg/day increment in vitamin C intake, 0.96 (95% CI = 0.94–0.99) for each 30 IU/day increment in vitamin E intake, and 1.00 (95% CI = 0.88–1.14) for each 1 mg/day increment in β-carotene intake. Jayedi et al. (53) included 11 prospective cohort studies in linear and nonlinear dose–response analyses of dietary folate, vitamin B6, and vitamin B12. They found linear inverse associations of folate and vitamin B6 intake with CHD risk, whereas no clear association was observed for vitamin B12.

Compared with these studies, the present study evaluated a broader range of exposures and data types. In addition to dietary intake and supplement use, circulating biomarkers were included, and vitamins, antioxidants, and related fatty acids were analyzed separately. Dietary measures primarily reflect nutrient intake but are susceptible to recall and reporting errors. Circulating biomarkers reflect nutrient status after absorption and metabolism but may also vary according to the measurement method. Because these measures represent different aspects of nutritional exposure, they were analyzed in parallel rather than combined. For exposures with sufficient dose information, restricted cubic spline models were used to examine dose–response patterns that might not be captured by a linear estimate based on a fixed increment.

Dietary vitamin C provides an illustrative but inconclusive example. The linear model reported by Ye et al. (13) indicated little change in CHD risk for each 30 mg/day increment in intake (RR = 1.01, 95% CI = 0.99–1.02). In the present study, spline modeling of the category-specific data reported by Knekt et al. (43) yielded a statistically significant overall model test (*p* = 0.0031), and the test for nonlinearity was also statistically significant (*p* = 0.0057), providing evidence of a nonlinear pattern. In the original categorical data, the RR point estimates were 0.92 at 84 mg/day and 1.23 at 152 mg/day. However, these category-specific estimates should not be interpreted as establishing a protective effect at moderate intake or a reversal of the association at higher intake. The analysis was based on one multi-cohort pooled study, showed no statistical evidence of residual lack of fit according to the goodness-of-fit test (*p* = 0.2683), and did not include exactly the same studies as the meta-analysis by Ye et al. Therefore, the observed curve should be regarded as exploratory, and the difference between the two analyses cannot be attributed solely to the statistical model.

This difference was not observed for all nutrients. For dietary β-carotene, Ye et al. (13) reported an RR of 1.00 (95% CI = 0.88–1.14) for each 1 mg/day increment, and the present RR-based analysis likewise found neither a statistically significant overall dose–response association (*p* = 0.6859) nor evidence of nonlinearity (*p* for nonlinearity = 0.9208). For B vitamins, the study-specific ORs for circulating vitamin B6, folate, and vitamin B12 were 0.19, 0.40, and 1.20, respectively. The directions of these associations were broadly consistent with the dietary intake findings reported by Jayedi et al. (53). However, the magnitudes of the effect estimates cannot be compared directly because the exposure measures, study designs, and effect measures differed, and each estimate in the present study was derived from a single study. These comparisons suggest that differences between the present findings and previous meta-analyses may reflect the combined influence of the evidence base, population composition, exposure assessment, and statistical modeling.

### 4.3 Mechanism analysis

Previous epidemiological studies have linked circulating antioxidant levels to CHD risk (54, 55). In the present study, the pooled OR for circulating carotenoids was 0.64. The estimates for β-carotene (OR = 0.63) and lycopene (OR = 0.65) were derived from a single cohort study and were not pooled. Although their direction was consistent with the pooled carotenoid result, these associations require confirmation in further studies. Antioxidants may influence CHD risk through reduced oxidative stress, improved endothelial function, and modulation of vascular smooth muscle cell proliferation (56, 57). In early atherosclerosis, oxidized low-density lipoprotein (ox-LDL) plays a central role in endothelial damage and the subsequent formation of foam cells (58). Vitamins C and E, together with ubiquinol, may reduce ox-LDL generation and limit foam cell accumulation in plaques by neutralizing free radicals and interrupting the chain reaction of lipid peroxidation (59, 60). Beyond this direct effect, some antioxidants—carotenoids in particular—also upregulate the Nrf2 pathway, which drives the production of endogenous antioxidant enzymes such as glutathione peroxidase and superoxide dismutase, and this may strengthen endothelial resistance to oxidative damage (61). Antioxidants can also interfere with the inflammatory signaling between endoplasmic reticulum stress and the NF-κB pathway, reducing vascular endothelial inflammation and slowing atherogenesis (62). At the functional level, these compounds may improve endothelial health and reduce thrombotic tendency by raising the availability of nitric oxide (NO), preserving vasodilatory capacity and limiting thrombus formation (63). These proposed mechanisms provide biological plausibility for the observed associations but do not establish that higher circulating antioxidant concentrations are cardioprotective or that antioxidant supplementation prevents CHD.

Certain fat-soluble vitamins may influence cardiovascular pathways through specific receptors rather than classical antioxidant mechanisms. Vitamin D signaling participates in calcium-phosphorus homeostasis and acts through vitamin D receptors (VDRs) expressed in cardiovascular tissues and immune cells. Experimental and mechanistic studies suggest that VDR-related pathways may modulate renin-angiotensin-aldosterone system activity, vascular smooth muscle remodeling, macrophage polarization, and NLRP3 inflammasome activation (64–66). These proposed mechanisms provide biological plausibility for the inverse association observed in the pooled HR analysis (HR = 0.79), but they do not establish causality or demonstrate that maintaining a particular serum 25(OH)D concentration prevents CHD. A separate case–control study reported a positive association at serum 25(OH)D concentrations ≥222.5 nmol/L (OR = 3.18), raising the possibility that its biological effects may differ at very high concentrations. However, this estimate was derived from a single small case–control study conducted in a male Indian population (n = 213, including 143 CHD cases) and should not be interpreted as evidence of a concentration-dependent reversal in risk unless confirmed in larger, independent, and sex-balanced samples.

Individual studies indicated inverse associations of circulating vitamin B6 and folate with CHD risk; the direction-harmonized OR was 0.19 for vitamin B6 and 0.40 for folate, whereas vitamin B12 showed no significant association. These associations may be related to the shared roles of B vitamins as cofactors in homocysteine (Hcy) metabolism. Folate, in its active 5-methyltetrahydrofolate form, donates methyl groups via methionine synthase (a B12-dependent enzyme) to convert Hcy back to methionine, while pyridoxal phosphate (PLP), the active form of vitamin B6, serves as a cofactor for cystathionine β-synthase, channeling Hcy into the transsulfuration pathway for conversion to cysteine. When B-vitamin intake is inadequate, circulating Hcy accumulates and damages the cardiovascular system through several routes (67, 68). Hcy undergoes auto-oxidation to yield hydrogen peroxide and superoxide, which together promote the oxidative modification of LDL and worsen ox-LDL-mediated endothelial injury (69). Hcy also promotes eNOS uncoupling and raises circulating levels of asymmetric dimethylarginine (ADMA), reducing NO availability and impairing endothelium-dependent vasodilation (70). In addition, Hcy activates coagulation factor V while inhibiting protein C and thrombin-regulatory proteins, pushing the haemostatic balance toward a pro-thrombotic state, and it can trigger endoplasmic reticulum stress and the unfolded protein response in endothelial cells, eventually leading to endothelial apoptosis (71). These pathways provide biological plausibility for an association between B vitamins and CHD risk but do not establish the magnitude or causality of the single-study estimates. Because each biomarker-specific estimate was derived from only one study, its consistency across populations could not be assessed, and residual confounding, selection bias, or chance may have influenced the results. The null finding for vitamin B12 may reflect the low prevalence of severe deficiency in the study population, but this explanation also requires confirmation.

The subtype-specific pooled estimates suggested that the directions of association with CHD risk differed across fatty acids. Limited pooled evidence from two studies indicated that myristic acid (C14:0; OR = 1.31), palmitic acid (C16:0; OR = 1.52), and palmitoleic acid (C16:1; OR = 1.41) were associated with higher CHD risk, whereas linoleic acid (C18:2; OR = 0.69) was associated with lower risk and DGLA (C20:3n-6; OR = 1.26) was associated with higher risk. Stearic acid (C18:0) showed no statistically significant association. Mechanistic studies suggest that shorter-chain saturated fatty acids may activate TLR4-related inflammatory pathways and promote ceramide synthesis, whereas stearic acid can be converted to oleic acid through Δ9-desaturase activity (72, 73). Linoleic acid may be incorporated into membrane phospholipids and influence PPAR-α-mediated fatty acid metabolism and circulating LDL-C levels (74, 75). DGLA is an intermediate in the arachidonic acid pathway and can be converted to arachidonic acid, a precursor of inflammatory and thrombotic eicosanoids (76). These mechanisms provide possible explanations for the observed associations but do not establish that the fatty acids have causal protective or harmful effects. Although n-3 PUFAs may influence arachidonic acid metabolism and the production of pro-resolving mediators (77), the pooled estimates for α-linolenic acid, EPA, DHA, and DPA were not statistically significant and their 95% CIs included 1.0. The available evidence therefore does not establish a clear association between circulating n-3 PUFA levels and CHD risk.

The direction of several point estimates varied across exposure ranges, but the available data are insufficient to interpret these patterns as established reversals of protective effects or evidence of metabolic saturation. For dietary vitamin C, the five-knot model showed a statistically significant overall dose–response association (*p* = 0.0031) and evidence of nonlinearity (*p* = 0.0057), while the goodness-of-fit test was not statistically significant (*p* = 0.2683). However, the analysis was based on only one cohort study with five exposure categories. Therefore, these variations should not be interpreted as evidence that moderate intake reduces CHD risk or that higher intake reverses the direction of the association; the range of 84–110 mg/day should be regarded only as a study-specific observation. For plasma ubiquinol, the point estimate changed from OR = 0.61 (95% CI = 0.33–1.13) at 0.60 μmol/L to OR = 1.22 (95% CI = 0.62–2.40) in the highest concentration category. However, neither the overall dose–response association (*p* = 0.1816) nor the test for nonlinearity (*p* = 0.0889) was statistically significant. These findings do not establish a reversal of a protective effect. The OR for blood α-tocopherol was 2.09 in the highest concentration category, but the 95% CI included 1.0 (95% CI = 0.96–4.52), precluding a conclusion of increased CHD risk. Serum 25(OH)D showed an inverse association in one HR analysis (HR = 0.79) and a positive association at an extremely high concentration in a separate OR analysis (OR = 3.18). Because these estimates were obtained from different studies and effect measures, they do not define a common clinical threshold.

If these exposure-risk patterns are confirmed in future studies, two potential mechanisms may be considered. First, once antioxidant levels surpass the capacity of endogenous reducing systems such as glutathione and NADPH, the resulting semi-oxidized intermediates (e.g., ascorbyl radicals, tocopheroxyl radicals) can react with transition metals or oxygen to generate reactive oxygen species (ROS), shifting the net effect from antioxidant to pro-oxidant (78). Second, for endocrine vitamins such as vitamin D, excessive VDR activation disturbs calcium-phosphorus balance, favoring calcium deposition in vessel walls and microcalcification within plaques, which may convert the original anti-inflammatory benefit into a plaque-destabilizing effect (79–81). These mechanisms provide hypotheses for further investigation, but the available evidence neither confirms the observed curve patterns nor identifies specific doses.

### 4.4 Clinical significance

The clinical interpretation of the observed associations requires careful consideration of the uncertainty surrounding the effect estimates. The available evidence was derived mainly from observational studies, and several estimates had wide 95% CIs or included the null value. For example, the estimates for blood vitamin E after sensitivity analysis (OR = 1.35, 95% CI = 0.99–1.85) and blood α-tocopherol in the highest concentration category (OR = 2.09, 95% CI = 0.96–4.52) were not statistically significant. These point estimates therefore describe only the direction of the observed associations and should not be regarded as established protective or harmful effects.

Accordingly, the current evidence does not establish CHD-specific nutrient intake targets, support high-dose antioxidant supplementation, or define biomarker thresholds for CHD prevention. Evidence from randomized trials has not shown a consistent reduction in cardiovascular events with vitamin or antioxidant supplementation (82). Although personalized nutritional assessment remains an area of research interest (83), current preventive practice should continue to emphasize healthy dietary patterns (84) and the identification and correction of clinically documented nutrient deficiencies when indicated. Whether biomarker-guided supplementation improves cardiovascular outcomes requires confirmation in prospective studies and randomized controlled trials.

### 4.5 Limitations and future prospects

Some caution is needed when interpreting these findings, given the heterogeneity inherent in cross-study comparisons and the limitations specific to this study. Moderate or substantial between-study heterogeneity was observed in the pooled analyses of multivitamin use, blood vitamin E, stearic acid, and DPA, indicating variation in the results across studies. Subgroup and sensitivity analyses suggested that differences in sex composition, CHD outcome ascertainment, and biomarker measurement methods may have contributed to this heterogeneity. However, formal endpoint-based subgroup comparisons were not feasible because most endpoint categories were represented by only one independent study. Moreover, the endpoint-based sensitivity analyses for serum 25(OH)D and blood vitamin E were based on limited numbers of studies or publications and should therefore be regarded as exploratory. Although excluding individual studies did not change the statistical significance of the pooled results, some point estimates shifted appreciably, indicating limited stability in the magnitude of the effects. Accordingly, these pooled estimates mainly reflect average effects across different study settings, and their applicability to specific populations should be assessed with caution.

Dietary patterns and baseline cardiovascular risk vary across regions and may influence the association between nutrient exposure and CHD risk (84). Evidence on circulating 25(OH)D was unevenly distributed across racial and ethnic groups and came predominantly from White populations, with only one study each in Asian and Black populations. The applicability of the pooled estimate for circulating 25(OH)D to other racial and ethnic groups therefore remains uncertain. Similarly, the elevated risk observed at serum 25(OH)D concentrations ≥222.5 nmol/L was based on a single small case–control study in men, and its generalizability to other populations remains unconfirmed. Although the goodness-of-fit test for dietary vitamin C was not statistically significant, the five-knot model was based on only five exposure categories from a single cohort study and therefore had limited residual degrees of freedom. The fitted pattern may consequently be sensitive to sparse categorical data and model specification, and the non-significant goodness-of-fit test should not be interpreted as proof of adequate model fit.

The confounders included in the fully adjusted models also varied across studies (Supplementary Table S3), reflecting differences in study design, data availability, and the analytic decisions of the original investigators. Because a uniform minimum set of confounders could not be required across all included studies, residual confounding by inconsistently adjusted factors, such as physical activity or lipid-lowering medication use, cannot be excluded. Dietary habits may also change during long-term follow-up, reducing the ability of a single baseline measurement to represent long-term nutritional exposure (85). Some included studies also report small event counts at the extremes of the exposure range, leading to wide uncertainty intervals for those estimates. Because all exposure-specific analyses included fewer than 10 independent studies or publications, visual assessment of funnel plot asymmetry and the two estimable Egger regression tests had limited statistical power. Therefore, publication bias could neither be assessed with confidence nor excluded. The included studies did not uniformly report separate effect estimates for food-derived and supplement-derived nutrient intake, and individual-level data were not available; a sub-analysis formally distinguishing these two exposure sources was therefore not feasible for most nutrients in this review. Findings for circulating B vitamins and several dietary or circulating antioxidants were each derived from a single study, preventing an assessment of their consistency across study populations. For these single-study exposures, including vitamin B6, a study-design-stratified sensitivity analysis could not be performed because only one study contributed to each categorical estimate; the magnitude of these estimates should therefore be interpreted with particular caution. The corresponding estimates may have been influenced by residual confounding, selection bias, or chance, and their reproducibility and applicability require further evaluation.

These limitations suggest that future prospective studies should collect nutritional assessments at several time points to better track changing exposure over time. The possible nonlinear patterns observed in this study require confirmation in larger prospective studies. Where feasible, randomized trials incorporating metabolomic and other multi-omics assessments may further clarify causality and inter-individual variation.

## 5 Conclusion

In conclusion, this meta-analysis of observational studies found that selected dietary and circulating vitamin, antioxidant, and fatty acid exposures were associated with CHD risk. Dose–response analyses suggested possible nonlinear patterns for some nutrients; however, several analyses, including that of dietary vitamin C, were based on single studies and therefore cannot define physiological thresholds or optimal intake ranges. These findings do not establish causality and should not be used to guide precision supplementation or high-dose antioxidant use for CHD prevention. Further prospective studies using standardized and repeated exposure measurements, together with appropriately designed randomized trials where relevant, are needed before clinical recommendations can be made.

## Data Availability

The original contributions presented in the study are included in the article/Supplementary material, further inquiries can be directed to the corresponding author/s.
