# Supplementary material for: Dietary intake and circulating levels of vitamins, antioxidants, and fatty acids in relation to coronary heart disease risk: a systematic review and dose–response meta-analysis
Source: Front Nutr. 2026 Oct 1;13:1920716. doi: 10.3389/fnut.2026.1920716 (PMC13668593; doi:10.3389/fnut.2026.1920716)
Supplement: Supplementary file 1 [file Table_1.DOCX]

**Supplementary Material**

***Dietary Intake and Circulating Levels of Vitamins, Antioxidants, and Fatty Acids in Relation to Coronary Heart Disease Risk: A Systematic Review and Dose-Response Meta-Analysis***

***Table of contents***

[**Supplementary Table 1 Search strategy: terms, databases and number of articles for review.	1**](#_Toc8771)

[**Supplementary Table 2.1 Customized Newcastle-Ottawa Scale (NOS) for Case-Control Studies	4**](#_Toc20603)

[**Supplementary Table 2.2 Customized Newcastle-Ottawa Scale (NOS) for Cohort Studies	7**](#_Toc22060)

[**Supplementary Table 2.3 Customized Agency for Healthcare Research and Quality (AHRQ) Checklist for Cross-Sectional Studies	9**](#_Toc23407)

[**Supplementary Table 3 Study characteristics	10**](#_Toc27920)

[**Supplementary Figure 1 Forest plots of the associations between various dietary intakes, blood biomarkers, and the risk of CHD	20**](#_Toc95)

[**Supplementary Figure 2 Sensitivity Analysis Diagram of Influencing Factors	25**](#_Toc15759)

[**Supplementary Figure 3 Dose-response curve plot	27**](#_Toc29073)

[**Supplementary Figure 4 Exploratory funnel plots for the exposure-specific meta-analyses	34**](#_Toc24582)

[**Supplementary Figure 5 Exploratory Egger regression plots for the two estimable analyses	41**](#_Toc19879)

**Supplementary Table 1** Search strategy: terms, databases and number of articles for review.

| **Databases** | **Strategy** | **Number of hits** |
| --- | --- | --- |
| PubMed  Date (publication):  Inception-2026/2/2 | ("Coronary Disease"[Mesh] OR "Coronary Artery Disease"[Mesh] OR "Myocardial Ischemia"[Mesh] OR "Myocardial Infarction"[Mesh] OR "Acute Coronary Syndrome"[Mesh] OR "Angina Pectoris"[Mesh]) OR ("Coronary Heart Disease"[Title/Abstract] OR "Coronary Disease"[Title/Abstract] OR "Coronary Artery Disease"[Title/Abstract] OR "Ischemic Heart Disease"[Title/Abstract] OR "Ischaemic Heart Disease"[Title/Abstract] OR "Myocardial Infarction*"[Title/Abstract] OR "Myocardial Ischemia"[Title/Abstract] OR "Acute Coronary Syndrome"[Title/Abstract] OR "Angina"[Title/Abstract] OR "Heart Attack*"[Title/Abstract] OR "Coronary Arteriosclerosis"[Title/Abstract] OR "Coronary Atherosclerosis"[Title/Abstract] OR "CAD"[Title/Abstract] OR "CHD"[Title/Abstract] OR "ACS"[Title/Abstract]) AND ((((((((((((((((((((((((((((((((vitamin[Title/Abstract]) OR (antioxidant[Title/Abstract])) OR (vitamin A[Title/Abstract])) OR (vitamin B[Title/Abstract])) OR (vitamin B12[Title/Abstract])) OR (ascorbic acid[Title/Abstract])) OR (vitamin D[Title/Abstract])) OR (multivitamins[Title/Abstract])) OR (vitamin E[Title/Abstract])) OR (retinoid[Title/Abstract])) OR (ubiquinol[Title/Abstract])) OR (coenzyme Q10[Title/Abstract])) OR (benfotiamine[Title/Abstract])) OR (folic acid[Title/Abstract])) OR (α-lipoic acid[Title/Abstract])) OR (fatty acids[Title/Abstract])) OR (pyrogallol[Title/Abstract])) OR (thioctic acid[Title/Abstract])) OR (selenium[Title/Abstract])) OR (zinc[Title/Abstract])) OR (glutamine[Title/Abstract])) OR (cysteine[Title/Abstract])) OR (superoxide enzyme[Title/Abstract])) OR (glutathione[Title/Abstract])) OR (acetylcysteine[Title/Abstract])) OR (lycopene[Title/Abstract])) OR (astaxanthin[Title/Abstract])) OR (beta-carotene[Title/Abstract])) OR (carotenoids[Title/Abstract])) OR (quercetin[Title/Abstract])) OR (canthaxanthin[Title/Abstract])) OR (curcumin[Title/Abstract]) | 21,386 |

| **Databases** | **Strategy** | **Number of hits** |
| --- | --- | --- |
| Web of science  Date (publication):  Inception-2026/2/2 | TS=("Coronary Disease" OR "Coronary Artery Disease" OR "Myocardial Ischemia" OR "Myocardial Infarction*" OR "Acute Coronary Syndrome" OR "Angina Pectoris" OR "Coronary Heart Disease" OR "Ischemic Heart Disease" OR "Ischaemic Heart Disease" OR "Angina" OR "Heart Attack*" OR "Coronary Arteriosclerosis" OR "Coronary Atherosclerosis" OR "CAD" OR "CHD" OR "ACS") AND TS=(vitamin OR antioxidant OR "vitamin A" OR "vitamin B" OR "vitamin B12" OR "ascorbic acid" OR "vitamin D" OR multivitamins OR "vitamin E" OR retinoid OR ubiquinol OR "coenzyme Q10" OR benfotiamine OR "folic acid" OR "alpha-lipoic acid" OR "fatty acids" OR pyrogallol OR "thioctic acid" OR selenium OR zinc OR glutamine OR cysteine OR "superoxide enzyme" OR glutathione OR acetylcysteine OR lycopene OR astaxanthin OR "beta-carotene" OR carotenoids OR quercetin OR canthaxanthin OR curcumin) | 26,609 |
| **Databases** | **Strategy** | **Number of hits** |
| Cochrane Library  Date (publication):  Inception-2026/2/2 | #1：(Coronary Disease OR Coronary Artery Disease OR Myocardial Ischemia OR Myocardial Infarction* OR Acute Coronary Syndrome OR Angina Pectoris OR Coronary Heart Disease OR Ischemic Heart Disease OR Ischaemic Heart Disease OR Angina OR Heart Attack* OR Coronary Arteriosclerosis OR Coronary Atherosclerosis OR CAD OR CHD OR ACS):ti,ab,kw  #2：(vitamin OR antioxidant OR vitamin A OR vitamin B OR vitamin B12 OR ascorbic acid OR vitamin D OR multivitamins OR vitamin E OR retinoid OR ubiquinol OR coenzyme Q10 OR benfotiamine OR folic acid OR alpha-lipoic acid OR fatty acids OR pyrogallol OR thioctic acid OR selenium OR zinc OR glutamine OR cysteine OR superoxide enzyme OR glutathione OR acetylcysteine OR lycopene OR astaxanthin OR beta-carotene OR carotenoids OR quercetin OR canthaxanthin OR curcumin):ti,ab,kw  #3：#1 and #2 | 5,324 |

| **Databases** | **Strategy** | **Number of hits** |
| --- | --- | --- |
| Embase  Date (publication):  Inception-2026/2/2 | #1：('coronary artery disease'/exp OR 'heart muscle ischemia'/exp OR 'heart infarction'/exp OR 'acute coronary syndrome'/exp OR 'angina pectoris'/exp) OR ('coronary heart disease':ti,ab,kw OR 'coronary disease':ti,ab,kw OR 'coronary artery disease':ti,ab,kw OR 'ischemic heart disease':ti,ab,kw OR 'ischaemic heart disease':ti,ab,kw OR 'myocardial infarction*':ti,ab,kw OR 'myocardial ischemia':ti,ab,kw OR 'acute coronary syndrome':ti,ab,kw OR 'angina':ti,ab,kw OR 'heart attack*':ti,ab,kw OR 'coronary arteriosclerosis':ti,ab,kw OR 'coronary atherosclerosis':ti,ab,kw OR 'cad':ti,ab,kw OR 'chd':ti,ab,kw OR 'acs':ti,ab,kw)  #2：('vitamin':ti,ab,kw OR 'antioxidant':ti,ab,kw OR 'vitamin a':ti,ab,kw OR 'vitamin b':ti,ab,kw OR 'vitamin b12':ti,ab,kw OR 'ascorbic acid':ti,ab,kw OR 'vitamin d':ti,ab,kw OR 'multivitamins':ti,ab,kw OR 'vitamin e':ti,ab,kw OR 'retinoid':ti,ab,kw OR 'ubiquinol':ti,ab,kw OR 'coenzyme q10':ti,ab,kw OR 'benfotiamine':ti,ab,kw OR 'folic acid':ti,ab,kw OR 'α-lipoic acid':ti,ab,kw OR 'fatty acids':ti,ab,kw OR 'pyrogallol':ti,ab,kw OR 'thioctic acid':ti,ab,kw OR 'selenium':ti,ab,kw OR 'zinc':ti,ab,kw OR 'glutamine':ti,ab,kw OR 'cysteine':ti,ab,kw OR 'superoxide enzyme':ti,ab,kw OR 'glutathione':ti,ab,kw OR 'acetylcysteine':ti,ab,kw OR 'lycopene':ti,ab,kw OR 'astaxanthin':ti,ab,kw OR 'beta-carotene':ti,ab,kw OR 'carotenoids':ti,ab,kw OR 'quercetin':ti,ab,kw OR 'canthaxanthin':ti,ab,kw OR 'curcumin':ti,ab,kw)  #3：('case control study'/de OR 'clinical trial'/de OR 'cohort analysis'/de OR 'cross-sectional study'/de OR 'major clinical study'/de OR 'observational study'/de OR 'prospective study'/de OR 'questionnaire'/de OR 'retrospective study'/de)  #4：#1 AND #2 AND #3 | 12,286 |

**Supplementary Table 2.1** Customized Newcastle-Ottawa Scale (NOS) for Case-Control Studies

| **First author, year** | **Selection** | | | | **Comparability** | | **Exposure** | | | **Total Score** |
| --- | --- | --- | --- | --- | --- | --- | --- | --- | --- | --- |
|  | 1 | 2 | 3 | 4 | 1 | | 1 | 2 | 3 |  |
| **Tavani,1997** | 1 | 1 | 1 | 1 | 1 | 1 | 1 | 1 | 1 | 9 |
| **Tavani,2006** | 1 | 1 | 1 | 1 | 1 | 1 | 1 | 1 | 1 | 9 |
| **Holmberg,2017** | 1 | 1 | 1 | 1 | 1 | 1 | 1 | 1 | 1 | 9 |
| **Qi,2017** | 1 | 1 | 1 | 1 | 1 | 1 | 1 | 1 | 1 | 9 |
| **Roy,2015** | 1 | 1 | 0 | 1 | 1 | 1 | 1 | 1 | 1 | 8 |
| **Hense,1993** | 1 | 1 | 1 | 1 | 1 | 0 | 1 | 1 | 1 | 8 |
| **Kok,1989** | 1 | 1 | 1 | 1 | 1 | 1 | 1 | 1 | 0 | 8 |
| **Pancharuniti,1994** | 1 | 1 | 1 | 1 | 1 | 1 | 1 | 1 | 1 | 9 |
| **Page,2009** | 1 | 1 | 1 | 1 | 1 | 1 | 1 | 1 | 1 | 9 |
| **Naidoo,2012** | 1 | 1 | 1 | 1 | 1 | 1 | 1 | 1 | 1 | 9 |
| **Riemersma,1991** | 0 | 1 | 1 | 1 | 1 | 1 | 1 | 1 | 1 | 8 |
| **Rajasree,2001** | 1 | 1 | 0 | 1 | 1 | 1 | 1 | 1 | 1 | 8 |
| **Chei,2018** | 1 | 1 | 1 | 1 | 1 | 1 | 1 | 1 | 1 | 9 |
| **Simon,1995** | 1 | 1 | 1 | 1 | 0 | 1 | 1 | 1 | 1 | 8 |
| **Acharya,2021** | 1 | 1 | 1 | 1 | 1 | 1 | 1 | 1 | 1 | 9 |

Note: A study can be awarded a maximum of one star for each numbered item within the Selection and Exposure categories. A maximum of two stars can be given for Comparability.

Selection

1)Is the Case Definition Adequate?

a) Requires some independent validation

b) Record linkage or self-report with no reference to primary record

c)No description

2)Representativeness of the Cases

a)All eligible cases with outcome of interest over a defined period of time, all cases in a defined catchment area, all cases in a defined hospital or clinic, group of hospitals, health maintenance organisation, or an appropriate sample of those cases.

b)Not satisfying requirements in part (a) ,or not stated.

3)Selection of Controls

This item assesses whether the control series used in the study is derived from the same population as the cases and essentially would have been cases had the outcome been present.

a)Community controls (i.e. same community as cases and would be cases if had outcome)

b)Hospital controls, within same community as cases (i.e. not another city) but derived from a hospitalised population

c)No description

4)Definition of Controls

a)If cases are first occurrence of outcome, then it must explicitly state that controls have no history of this outcome. If cases have new (not necessarily first) occurrence of outcome, then controls with previous occurrences of outcome of interest should not be excluded.

b)No mention of history of outcome

Comparability

1)Comparability of Cases and Controls on the Basis of the Design or Analysis

A maximum of 2 stars can be allotted in this category

Either cases and controls must be matched in the design and/or confounders must be adjusted for in the analysis. Statements of no differences between groups or that differences were not statistically significant are not sufficient for establishing comparability. Note: If the odds ratio for the exposure of interest is adjusted for the confounders listed, then the groups will be considered to be comparable on each variable used in the adjustment.

There may be multiple ratings for this item for different categories of exposure (e.g. ever vs. never, current vs. previous or never)

Age = , Other controlled factors =

Exposure

1)Ascertainment of exposure

a) secure record ☆

b) structured interview where blind to case/control status ☆

c) interview not blinded to case/control status d) written self report or medical record only e) no description

2)Same method of ascertainment for cases and controls

a) yes ☆

b) no

3)Non-Response rate

a) same rate for both groups ☆

b) non respondents described

c) rate different and no designation

**Supplementary Table 2.2** Customized Newcastle-Ottawa Scale (NOS) for Cohort Studies

| **First author, year** | **Selection** | | | | **Comparability** | | **Outcome** | | | **Total Score** |
| --- | --- | --- | --- | --- | --- | --- | --- | --- | --- | --- |
|  | 1 | 2 | 3 | 4 | 1 | | 1 | 2 | 3 |  |
| **Michos,2015** | 1 | 1 | 1 | 1 | 1 | 1 | 1 | 1 | 1 | 9 |
| **Sun,2011** | 0 | 1 | 1 | 1 | 1 | 1 | 1 | 1 | 1 | 8 |
| **Harvey,2018** | 1 | 1 | 1 | 1 | 1 | 1 | 1 | 1 | 1 | 9 |
| **Rimm,1993** | 1 | 1 | 1 | 1 | 1 | 1 | 1 | 0 | 1 | 8 |
| **Karppi,2011** | 1 | 1 | 1 | 1 | 1 | 1 | 1 | 1 | 1 | 9 |
| **Rautiainen,2015** | 1 | 1 | 1 | 1 | 1 | 1 | 1 | 1 | 1 | 9 |
| **Rautiainen_2016** | 1 | 1 | 1 | 1 | 1 | 1 | 1 | 1 | 1 | 9 |
| **Perna,2013** | 1 | 1 | 1 | 1 | 1 | 1 | 1 | 1 | 1 | 9 |
| **Huang,2019** | 0 | 1 | 1 | 1 | 1 | 1 | 1 | 1 | 1 | 8 |
| **Knekt,2004** | 1 | 1 | 1 | 1 | 1 | 1 | 1 | 1 | 1 | 9 |
| **Pietinen,1997** | 1 | 1 | 1 | 1 | 1 | 1 | 1 | 1 | 1 | 9 |

Note: A study can be awarded a maximum of one star for each numbered item within the Selection and Outcome categories. A maximum of two stars can be given for Comparability

Selection

1) Representativeness of the exposed cohort

a) truly representative of the average characteristics in the community ☆ ¯

b) somewhat representative of the average characteristics in the community ☆ ¯

c) selected group of users eg nurses, volunteers

d) no description of the derivation of the cohort

2) Selection of the non-exposed cohort

a) drawn from the same community as the exposed cohort ☆ ¯

b) drawn from a different source

c) no description of the derivation of the non-exposed cohort

3) Ascertainment of exposure

a) secure record ☆ ¯

b) structured interview ☆ ¯

c) written self-report

d) no description

4) Demonstration that outcome of interest was not present at start of study

a) yes ☆ ¯

b) no

Comparability

1) Comparability of cohorts on the basis of the design or analysis

a) study controls for the most important factor☆ ¯

b) study controls for any additional factor ☆

Outcome

1) Assessment of outcome

a) independent blind assessment ☆ ¯

b) record linkage ☆ ¯

c) self-report

d) no description

2) Was follow-up long enough for outcomes to occur

a) yes ☆ ¯

b) no

3) Adequacy of follow up of cohorts

a) complete follow up - all subjects accounted for ☆ ¯

b) subjects lost to follow up unlikely to introduce bias - small number lost - > 90% follow up, or description provided of those lost) ☆ ¯

c) follow up rate < 90% (select an adequate %) and no description of those lost

d) no statement

**Supplementary Table 2.3** Customized Agency for Healthcare Research and Quality (AHRQ) Checklist for Cross-Sectional Studies

| **First author, year** | 1 | 2 | 3 | 4 | 5 | 6 | 7 | 8 | 9 | 10 | 11 | **Total Score** |
| --- | --- | --- | --- | --- | --- | --- | --- | --- | --- | --- | --- | --- |
| **Li,2024** | 1 | 1 | 1 | 1 | 0 | 1 | 1 | 1 | 1 | 1 | 1 | 10 |

Note: A study is awarded a score of 1 for each numbered item if it meets the specified criteria; otherwise, it scores 0. The methodological quality of cross-sectional studies was assessed using the 11-item checklist recommended by the Agency for Healthcare Research and Quality (AHRQ). Total scores range from 0 to 11 (0-3: low quality; 4-7: moderate quality; 8-11: high quality).

1) Define source of information (survey, record review): Explicitly stated.
2) List inclusion and exclusion criteria for exposed and unexposed subjects: Explicitly stated.
3) Indicate time period used for identifying patients: Explicitly stated.
4) Indicate whether or not subjects were consecutive if not population-based: Consecutive subjects or population-based probability sampling utilized.
5) Indicate if evaluators of subjective components of study were masked: Personnel conducting interviews or blood assays were blinded to the subjects' disease status.
6) Describe any assessments undertaken for quality assurance purposes: Quality control measures described.
7) Explain any patient exclusions from analysis: Reasons for exclusions clearly explained.
8) Describe how confounding was assessed and/or controlled: Study controls for the most important confounding factors in the statistical analysi.
9) Explain how missing data were handled in the analysis: Handling method explicitly described.
10) Summarize patient response rates and completeness of data collection: Response rate, participation rate, or completeness of data reported.
11) Clarify what follow-up, if any, was expected: Not applicable (since cross-sectional studies assess exposure and outcome simultaneously, this item is conventionally awarded 1 point if the cross-sectional design is explicitly stated and justified).

**Supplementary Table 3** Study characteristics

| **Author, Year** | **Design** | **Region** | **Sex / Population** | **Age (years)** | **Coronary outcome** | **N / Cases** | **Follow-up (y)** | **Exposures** | **Assessment** | **Adjusted Confounders** |
| --- | --- | --- | --- | --- | --- | --- | --- | --- | --- | --- |
| Michos, 2015 | cohort | US | Overall | 57 | CHD | 11,945 / 1,230 | 20 | blood_25(OH)D | Serum | Age, sex, race, BMI, education, physical activity, and smoking status. |
| Knekt, 2004 | cohort | US & Europe | Overall | ＞35 | Major CHD | 293,172 / 4,647 | 10 | Dietary Vit C, Vit E, Carotenes | Food frequency questionnaire (FFQ) | Age, sex, smoking, BMI, education, physical activity, alcohol, and total energy intake |
| Li, 2024 | Cross-sectional | US | Overall | 47.4 | CHD | 33,640 / 1,429 | N/A | Dietary Vit E | 24-h dietary recall | Age, sex, education level, race, family income, BMI, drinking status, smoking status, hypertension and diabetes |
| Tavani, 1997 | Case-control | Italy | Women | 18-74 | Nonfatal AMI | 1,302 / 433 | N/A | Dietary β-Carotene, Retinol | Structured questionnaire | Age, education, body mass index, smoking, alcohol consumption, coffee intake, menopausal status, hormone replacement therapy, history of diabetes, hypertension, and hyperlipidemia |

| **Author, Year** | **Design** | **Region** | **Sex / Population** | **Age (years)** | **Coronary outcome** | **N / Cases** | **Follow-up (y)** | **Exposures** | **Assessment** | **Adjusted Confounders** |
| --- | --- | --- | --- | --- | --- | --- | --- | --- | --- | --- |
| Harvey, 2018 | cohort | UK | Overall | 50-63 | IHD/MI | 475,255 / 10,513 | 7 | Vitamin D supplement | Touch-screen questionnaire | Age, BMI, smoking, family history of cardiac disease, alcohol, educational level, vigorous physical activity, systolic blood pressure, diabetes medications and cholesterol medications |
| Tavani,  2006 | Case-control | Italy | Overall | 18-79 | AMI | 1,442 / 760 | N/A | Dietary β-Carotene, Lycopene, Retinol | FFQ | Age,sex, study, education, smoking, alcohol, coffee, non-alcohol total calorie intake, body mass index, physical activity, cholesterol level, diabetes, hyperlipidemia, hypertension and family history of AMI |
| Holmberg,2017 | Nested case-control | Sweden | Men | Adult | IHD | 552 / 276 | N/A | Blood 25(OH)D | LC-MS/MS (Serum) | Age, month of blood sampling (season), and other baseline cardiovascular covariates |

| **Author, Year** | **Design** | **Region** | **Sex / Population** | **Age (years)** | **Coronary outcome** | **N / Cases** | **Follow-up (y)** | **Exposures** | **Assessment** | **Adjusted Confounders** |
| --- | --- | --- | --- | --- | --- | --- | --- | --- | --- | --- |
| Qi, 2017 | Nested case-control | US | Women | 43-79 | CHD | 957 / 382 | 20 | Blood 25(OH)D | Serum sample | Age, smoking, fasting status, time/season of blood draw, BMI, physical activity, alcohol intake, diet quality, and family history of CHD |
| Roy,2015 | Case-control | India | Overall | 25-75 | AMI | 240 / 120 | N/A | Blood 25(OH)D | Serum analysis | Age, sex, BMI, smoking, hypertension, diabetes, and dyslipidemia |
| Pietinen,1997 | Cohort | Finland | Men (Smokers) | 50-69 | CHD | 21,930 / 1,399 | 6.1 | Dietary Omega-3, SFA, and TFA | Validated dietary questionnaire | Age, supplement group, total energy, fiber, total fat, dietary cholesterol, smoking, BMI, blood pressure, blood lipids, physical activity, education, and history of CHD |
| Hense,1993 | nested case-control | Germany | Overall | 25–64 | MI | 230 / 46 | N/A | Blood Vitamin E | Serum analysis | Age, sex, total cholesterol, smoking, alcohol consumption, and HDL cholesterol |

| **Author, Year** | **Design** | **Region** | **Sex / Population** | **Age (years)** | **Coronary outcome** | **N / Cases** | **Follow-up (y)** | **Exposures** | **Assessment** | **Adjusted Confounders** |
| --- | --- | --- | --- | --- | --- | --- | --- | --- | --- | --- |
| Karppi, 2011 | Cohort | Finland | Men | 42–60 | AMI | 1,031 / 194 | 10.6 | Blood Lycopene, β-carotene, Retinol | Serum analysis | Age, examination year, alcohol consumption, smoking, BMI, systolic blood pressure, LDL cholesterol, HDL cholesterol, diabetes, and family history of coronary heart disease |
| Kok, 1989 | Case-control | Netherlands | Overall | ＜75 | AMI | 168 / 84 | N/A | Blood Vitamin B6 (PLP) | Serum analysis | Age, sex, smoking, serum cholesterol, blood pressure, and BMI |
| Rautiainen，2015 | Cohort | US | Women | ≥45 | CAD | 37,193 / 1,435 | 16.2 | Multivitamin use | Questionnaires and FFQ | Age, BMI, smoking, vigorous exercise, menopausal/HRT status, alcohol, hypertension, hypercholesterolemia, diabetes, parental MI, and dietary intake (fruit and vegetables, energy, fiber, saturated fat, and n-3 fatty acids). |

| **Author, Year** | **Design** | **Region** | **Sex / Population** | **Age (years)** | **Coronary outcome** | **N / Cases** | **Follow-up (y)** | **Exposures** | **Assessment** | **Adjusted Confounders** |
| --- | --- | --- | --- | --- | --- | --- | --- | --- | --- | --- |
| Rautiainen, 2016 | Cohort | US | Men | ≥40 | IHD | 18,530 / 1,706 | 12.2 | Multivitamin use | Baseline questionnaire | Age (continuous), randomized treatment assignments (aspirin, β-carotene), smoking status, physical activity, alcohol intake, body mass index (BMI), history of hypertension, history of high cholesterol, history of diabetes, and parental history of MI before 60 y of age |
| Pancharuniti,  1994 | Case-control | US | Men | 30-50 | CAD | 209 / 101 | N/A | Blood Folate, Blood Vitamin B-12 | Fasting blood sample (Radioimmunoassay) | Age, sex, race, smoking, hypertension, diabetes mellitus, total cholesterol, high-density lipoprotein (HDL) cholesterol, and family history of coronary artery disease |

| **Author, Year** | **Design** | **Region** | **Sex / Population** | **Age (years)** | **Coronary outcome** | **N / Cases** | **Follow-up (y)** | **Exposures** | **Assessment** | **Adjusted Confounders** |
| --- | --- | --- | --- | --- | --- | --- | --- | --- | --- | --- |
| Page,2009 | Nested case-control | US | Women | 60.6 ± 7.2 | MI | 402 / 144 | N/A | Blood Vitamin B6 (PLP) | Plasma analysis | Age, smoking status, date of blood draw, fasting status, BMI, physical activity, alcohol intake, history of hypertension, history of diabetes, family history of MI, menopausal status, and postmenopausal hormone use |
| Naidoo,2012 | Nested case-control | Singapore | Overall  (Singapore Chinese) | 45-74 | AMI | 699 / 233 | N/A | Blood α-Tocopherol, Blood Ubiquinol | Plasma analysis (HPLC) | Age at blood collection, gender, dialect group, year of interview, year of blood draw, total cholesterol, triglycerides, cigarette smoking, BMI, education, moderate-intensity physical activity, strenuous sports, strenuous work activity, alcohol consumption, plasma β-cryptoxanthin, plasma lutein, hypertension, and diabetes |
| **Author, Year** | **Design** | **Region** | **Sex / Population** | **Age (years)** | **Coronary outcome** | **N / Cases** | **Follow-up (y)** | **Exposures** | **Assessment** | **Adjusted Confounders** |
| Riemersma,1991 | Case-control | UK | Men | 35-54 | Angina | 504 / 110 | N/A | Blood Vitamin E, Vitamin C,Blood Total Carotene | Plasma analysis | Age, smoking, blood pressure, total cholesterol, triglycerides, and relative weight |
| Perna,2013 | Cohort | Germany | Overall | 50-74 | CHD | 7,709 / 460 | 8 | Blood 25(OH)D | Serum analysis | Age, sex, season of blood draw, body mass index (BMI), smoking status, physical activity, alcohol consumption, education level, history of diabetes, history of hypertension, and history of hypercholesterolemia |
| Rajasree,2001 | Case-control | India | Men | 45–65 | IHD | 213 / 143 | N/A | Blood 25(OH)D | Serum analysis | Age, body mass index (BMI), diabetes, hypertension, smoking, alcoholism, family history of IHD, and sedentary habits |

| **Author, Year** | **Design** | **Region** | **Sex / Population** | **Age (years)** | **Coronary outcome** | **N / Cases** | **Follow-up (y)** | **Exposures** | **Assessment** | **Adjusted Confounders** |
| --- | --- | --- | --- | --- | --- | --- | --- | --- | --- | --- |
| Chei,2018 | Nested case-control | Japan | Overall | 40-85 | CAD | 608 / 152 | 11.0 (Median) | Blood fatty acids | Serum analysis (Gas-liquid chromatography) | Age, sex, community, year of serum storage, fasting status, BMI, cigarette smoking status, and current alcohol intake |
| Simon,1995 | Nested case-control | US | Men | 35-57 | CHD | 188 / 94 | 6.9 (Average) | Blood fatty acids | Serum analysis (Gas-liquid chromatography) | Age, date of randomization, clinical center, tobacco use, diastolic blood pressure, and plasma cholesterol level |
| Acharya,2021 | nested case-control | US | Overall | ＞18 | MI | 19,092 / NR | 3.6–4.9 (Mean) | Blood 25(OH)D | Serum analysis | Age, sex,BMI,hypertension, diabetes mellitus, coronary artery disease, congestive heart failure, peripheral vascular disease, chronic kidney disease, chronic obstructive pulmonary disease, smoking, aspirin, statin, beta blockers, and low-density lipoprotein (LDL) |

| **Author, Year** | **Design** | **Region** | **Sex / Population** | **Age (years)** | **Coronary outcome** | **N / Cases** | **Follow-up (y)** | **Exposures** | **Assessment** | **Adjusted Confounders** |
| --- | --- | --- | --- | --- | --- | --- | --- | --- | --- | --- |
| Huang,2019 | Cohort | China& Denmark | Overall | 53.2–58.5 (Mean) | IHD | 49,457 / 10,386 | NR | Blood 25(OH)D | Plasma analysis (Immunoassay) | Age, sex, smoking status, alcohol intake, physical activity, SBP, season, and region/latitude |
| Sun, 2011 | Cohort | US | Overall | 49.3–55.3 (Mean range) | CHD | 118,864 / NR | 20-22 | Dietary Vitamin D | FFQ | Age, BMI, physical activity, smoking status, alcohol consumption, family history of myocardial infarction, aspirin use, multivitamin use, menopausal status and hormone use (in women), history of hypertension, high cholesterol, diabetes, total energy intake, and dietary intakes of calcium, retinol, trans fat, polyunsaturated-to-saturated fat ratio, cereal fiber, and glycemic load |
| Rimm, 1993 | Cohort | US | Men | 40–75 | CHD | 39,910 / 667 | 4 | Dietary Vitamin E | FFQ | questionnaire (FFQ)Age, smoking, BMI, alcohol intake, history of hypertension, history of high blood cholesterol, parental history of myocardial infarction before age 60, profession, total energy intake, and dietary intakes of vitamin C, carotene, saturated fat, polyunsaturated fat, cholesterol, and dietary fiber |

Abbreviations: CHD, coronary heart disease; IHD, ischemic heart disease; CAD, coronary artery disease; MI, myocardial infarction; AMI, acute myocardial infarction.

**Supplementary Figure 1 Forest plots of the associations between various dietary intakes, blood biomarkers, and the risk of CHD**

**Figure S1.1**: Forest plot of the association between multivitamin supplementation and the risk of CHD

**Figure S1.2:** Forest plot of the association between multivitamin supplementation and the risk of CHD in sex subgroups

**Figure S1.3:** Forest plot of the association between vitamin D supplementation and the risk of CHD in sex subgroups

**Figure S1.4:** Forest plot of the association between quantitative dietary vitamin D intake and the risk of CHD in sex subgroups

**Figure S1.5:** Forest plot of the association between blood vitamin E levels and the risk of CHD

**Figure S1.6:** Forest plot of the association between blood vitamin E levels and the risk of CHD in dose subgroups

**Figure S1.7:** Forest plot of the association between blood n-3 polyunsaturated fatty acid (n-3 PUFA) levels and the risk of CHD in subgroups

**Figure S1.8:** Forest plot of the association between blood B vitamin (B6, B12, and folate) levels and the risk of CHD

**Figure S1.9:** Forest plot of the association between dietary antioxidant intake levels and the risk of CHD

**Figure S1.10:** Forest plot of the association between blood antioxidant levels and the risk of CHD

**Supplementary Figure 2 Sensitivity Analysis Diagram of Influencing Factors**

**Figure S2.1:** Leave-one-out sensitivity analysis of the association between blood vitamin E levels and the risk of CHD

**Figure S2.2:** Leave-one-out sensitivity analysis of the association between blood stearic acid (C18:0) levels and the risk of CHD

**Figure S2.3:** Leave-one-out sensitivity analysis of the association between blood docosapentaenoic acid (DPA, C22:5) levels and the risk of CHD

**Supplementary Figure 3 Dose-response curve plot**

**Figure S3.1:** Dose-response curve of the association between dietary vitamin E intake and the risk of CHD

**Figure S3.2:** Dose-response curve of the association between dietary lycopene intake and the risk of CHD

**Figure S3.3:** Dose-response curve of the association between dietary total carotene intake and the risk of CHD

**Figure S3.4:** Dose-response curve of the association between dietary β-carotene intake and the risk of CHD (effect size: RR)

**Figure S3.5:** Dose-response curve of the association between dietary β-carotene intake and the risk of CHD (effect size: OR)

**Figure S3.6:** Dose-response curve of the association between dietary retinol intake and the risk of CHD

**Figure S3.7:** Dose-response curve of the association between dietary omega-3 fatty acid intake and the risk of CHD

**Figure S3.8:** Dose-response curve of the association between dietary saturated fatty acid intake and the risk of CHD

**Figure S3.9:** Dose-response curve of the association between dietary trans fatty acid intake and the risk of CHD

**Figure S3.10:** Dose-response curve of the association between serum 25(OH)D levels and the risk of CHD (effect size: HR)

**Figure S3.11:** Dose-response curve of the association between serum 25(OH)D levels and the risk of CHD (effect size: OR)

**Figure S3.12:** Dose-response curve of the association between blood α-tocopherol levels and the risk of CHD

**Figure S3.13:** Dose-response curve of the association between blood vitamin B6 (PLP) levels and the risk of CHD

**Figure S3.14:** Dose-response curve of the association between plasma ubiquinol concentration and the risk of CHD

**Supplementary Figure 4 Exploratory funnel plots for the exposure-specific meta-analyses**

Note: All funnel plots are presented for transparency only. Because each exposure-specific analysis included fewer than 10 independent studies or publications, visual assessment of funnel plot asymmetry had limited reliability and was not used to confirm or exclude publication bias.

**Figure S4.1:** Funnel plot of the association between multivitamin supplementation and the risk of CHD

**Figure S4.2:** Funnel plot of the association between serum 25-hydroxyvitamin D [25(OH)D] levels and the risk of CHD (effect size: HR)

**Figure S4.3:** Funnel plot of the association between serum 25-hydroxyvitamin D [25(OH)D] levels and the risk of CHD (effect size: OR)

**Figure S4.4:** Funnel plot of the association between blood vitamin E levels and the risk of CHD

**Figure S4.5:** Funnel plot of the association between blood carotene levels and the risk of CHD

**Figure S4.6:** Funnel plot of the association between dietary antioxidant intake and the risk of CHD

**Figure S4.7:** Funnel plot of the association between blood antioxidant levels and the risk of CHD

**Figure S4.8:** Funnel plot of the association between blood myristic acid (C14:0) levels and the risk of CHD

**Figure S4.9:** Funnel plot of the association between blood palmitic acid (C16:0) levels and the risk of CHD

**Figure S4.10:** Funnel plot of the association between blood stearic acid (C18:0) levels and the risk of CHD

**Figure S4.11:** Funnel plot of the association between blood palmitoleic acid (C16:1) levels and the risk of CHD

**Figure S4.12:** Funnel plot of the association between blood oleic acid (C18:1) levels and the risk of CHD

**Figure S4.13:** Funnel plot of the association between blood α-linolenic acid (ALA, C18:3 n-3) levels and the risk of CHD

**Figure S4.14:** Funnel plot of the association between blood eicosapentaenoic acid (EPA, C20:5 n-3) levels and the risk of CHD

**Figure S4.15:** Funnel plot of the association between blood docosapentaenoic acid (DPA, C22:5 n-3) levels and the risk of CHD

**Figure S4.16:** Funnel plot of the association between blood docosahexaenoic acid (DHA, C22:6 n-3) levels and the risk of CHD

**Figure S4.17:** Funnel plot of the association between blood linoleic acid (LA, C18:2 n-6) levels and the risk of CHD

**Figure S4.18:** Funnel plot of the association between blood dihomo-γ-linolenic acid (DGLA, C20:3 n-6) levels and the risk of CHD

**Figure S4.19:** Funnel plot of the association between blood arachidonic acid (AA, C20:4 n-6) levels and the risk of CHD

**Supplementary Figure 5. Exploratory Egger regression plots for the two estimable analyses**

**Figure S5.1:** Egger regression plot for the association between serum 25-hydroxyvitamin D [25(OH)D] levels and CHD risk (effect measure: HR).

**Note:** The Egger bias coefficient was 0.875 (95% CI = -2.482 to 4.232; P = 0.468). Because the model included only three publications contributing five non-overlapping estimates, the test had limited statistical power and is presented for transparency only.

**Figure S5.2:** Egger regression plot for the association between blood vitamin E levels and CHD risk (effect measure: OR).

**Note:** The Egger bias coefficient was −4.553 (95% CI = −31.126 to 22.019; P = 0.274). Because only three independent studies were included, the test had very limited statistical power and is presented for transparency only.
